# Proteomic profiling of formalin-fixed paraffine-embedded tissue reveals key proteins related to lung dysfunction in idiopathic pulmonary fibrosis

**DOI:** 10.3389/fonc.2023.1275346

**Published:** 2024-01-23

**Authors:** Anna Valeria Samarelli, Roberto Tonelli, Giulia Raineri, Giulia Bruzzi, Dario Andrisani, Filippo Gozzi, Alessandro Marchioni, Matteo Costantini, Luca Fabbiani, Filippo Genovese, Diego Pinetti, Linda Manicardi, Ivana Castaniere, Valentina Masciale, Beatrice Aramini, Luca Tabbì, Simone Rizzato, Stefania Bettelli, Samantha Manfredini, Massimo Dominici, Enrico Clini, Stefania Cerri

**Affiliations:** ^1^Laboratory of Cell Therapies and Respiratory Medicine, Department of Medical and Surgical Sciences for Children & Adults, University Hospital of Modena, Modena, Italy; ^2^Respiratory Disease Unit, Department of Medical and Surgical Sciences, University of Modena and Reggio Emilia, University Hospital of Modena, Modena, Italy; ^3^Pathology Institute, University of Modena and Reggio Emilia, University Hospital of Modena, Modena, Italy; ^4^Immunohistochemistry Lab, University of Modena and Reggio Emilia, University Hospital of Modena, Modena, Italy; ^5^Centro Interdipartimentale Grandi Strumenti (C.I.G.S.), University of Modena and Reggio Emilia, Modena, Italy; ^6^Laboratory of Cellular Therapy, Department of Medical and Surgical Sciences for Children & Adults, University Hospital of Modena and Reggio Emilia, Modena, Italy; ^7^Oncology Unit, University Hospital of Modena and Reggio Emilia, University of Modena and Reggio Emilia, Modena, Italy; ^8^Division of Thoracic Surgery, Department of Medical and Surgical Sciences-Diagnostic and Specialty Medicine (DIMEC) of the Alma Mater Studiorum, University of Bologna G.B. Morgagni-L. Pierantoni Hospital, Forlì, Italy; ^9^Molecular Pathology and Predictive Medicine Unit, Modena Cancer Center, University Hospital of Modena, Modena, Italy

**Keywords:** pulmonary fibrosis, mass spectrometry, molecular profiling, lung function decline, rare disease, IPF

## Abstract

**Introduction:**

Idiopathic pulmonary fibrosis (IPF) severely affects the lung leading to aberrant deposition of extracellular matrix and parenchymal stiffness with progressive functional derangement. The limited availability of fresh tissues represents one of the major limitations to study the molecular profiling of IPF lung tissue. The primary aim of this study was to explore the proteomic profiling yield of archived formalin-fixed paraffin-embedded (FFPE) specimens of IPF lung tissues.

**Methods:**

We further determined the protein expression according to respiratory functional decline at the time of biopsy. The total proteins isolated from 11 FFPE samples of IPF patients compared to 3 FFPE samples from a non-fibrotic lung defined as controls, were subjected to label-free quantitative proteomic analysis by liquid chromatography-mass spectrometry (LC-MS/MS) and resulted in the detection of about 400 proteins.

**Results:**

After the pairwise comparison between controls and IPF, functional enrichment analysis identified differentially expressed proteins that were involved in extracellular matrix signaling pathways, focal adhesion and transforming growth factor β (TGF-β) signaling pathways strongly associated with IPF onset and progression. Five proteins were significantly over- expressed in the lung of IPF patients with either advanced disease stage (Stage II) or impaired pulmonary function (FVC<75, DLCO<55) compared to controls; these were lymphocyte cytosolic protein 1 (LCP1), peroxiredoxin-2 (PRDX2), transgelin 2 (TAGLN2), lumican (LUM) and mimecan (OGN) that might play a key role in the fibrogenic processes.

**Discussion:**

Our work showed that the analysis of FFPE samples was able to identify key proteins that might be crucial for the IPF pathogenesis. These proteins are correlated with lung carcinogenesis or involved in the immune landscape of lung cancer, thus making possible common mechanisms between lung carcinogenesis and fibrosis progression, two pathological conditions at risk for each other in the real life.

## 1 Introduction

Idiopathic pulmonary fibrosis (IPF) is a rare, chronic, progressive, fibrosing interstitial lung disease (ILD) of still unknown etiology, with a median survival of 3 years from the time of diagnosis (1–3). Besides the promising results of two antifibrotic drugs (Pirfenidone and Nintedanib) in slowing down the respiratory functional decline of IPF patients, an improvement in mortality rate has not yet been achieved (4–6). Since the histological pattern is not exclusively associated with IPF but with other ILDs, a proven diagnosis of IPF may represent a challenge for physicians (3, 7). Many evidences suggest that IPF is a consequence of multiple interacting genetic, and environmental risk factors such as cigarettes smoking, metal, and silica dust or microbial agents (8–11), leading to repetitive local micro-injuries to aging alveolar epithelium, aberrant epithelial–fibroblast functional activity, induction of matrix-producing myofibroblasts, exaggerate extracellular matrix deposition, and distortion of lung architecture (12, 13). In addition, some genetic variants have also been associated with IPF onset and progression such as the mucin 5B (MUC5B) gene, involved in the maintenance of bronchoalveolar epithelial function (14–16), or TERT and TERC genes involved in the telomere length and maintenance (17–20). Notwithstanding, IPF remains a fatal disease, thus the identification of new biomarkers indicative of disease progression represents an unmet clinical need that has to be addressed (21, 22).

Over the past ten years, proteomic analysis through mass spectrometry (MS) has become an extremely sensitive tool for characterizing key proteins involved in the progression of IPF as a chronic disease, elucidating the molecular mechanisms and profile of patients (23–26). Therefore, the performance of MS proteomics has been maximized with spatially resolved proteomic to precisely delineate the molecular profile characterized by distinct protein compositions in the histologically defined regions of tissue in comparison to whole lung proteome (27–29). However, given the limitation of fresh tissues from patients with rare and chronic condition, the MS of archived tissues stored as formalin-fixed paraffin-embedded (FFPE) blocks have been widely and more easily used in research (30, 31). However, the MS analysis on FFPE blocks can be challenging due to the covalent crosslinking and low solubility of extracellular matrix (ECM) proteins that are aberrantly enriched in the lung tissue of patients (32).

Herein, we described for the first time the applicability and the performance of a protocol implying the liquid chromatography-mass spectrometry (LC-MS/MS) that allowed the isolation of total proteins from FFPE blocks of IPF patients, different from the recent application of Laser capture microdissection coupled mass spectrometry (LCM-MS) for spatially resolved analysis of FFPE from the same patients (33). Moreover, our study enabled the identification of differentially expressed proteins in IPF patients with severely impaired lung function, compared to non-fibrotic individuals, to possibly implement the knowledge on the disease progression.

## 2 Materials and methods

### 2.1 Ethics statement

Mass Spectrometry analyses on archived IPF samples were conducted after authorization by the ethics committee of the University of Modena and Reggio Emilia (557/2019/SPER/AOUMO).

### 2.2 Case identification and selection

Twenty patients with histologically confirmed idiopathic pulmonary fibrosis with UIP pattern, who underwent surgical lung biopsy between 2011 and 2020, in the process of being diagnosed towards a multidisciplinary team and according the most recent ATS/ERS/JRS/ALAT guidelines (1, 34), were considered eligible for our retrospective cohort study and therefore referred to as IPF patients from now on. We collected the clinicopathological data from electronic medical records present in the database of the Respiratory Disease Unit of University Hospital of Modena, where both deceased and living patients were included in the study. Of twenty patients, nineteen were eligible for the study with confirmed diagnosis of IPF being characterized by the histological and morphological pattern of Usual Interstitial Pneumonia (UIP). One patient was later diagnosed with pulmonary fibrosis secondary to systemic sclerosis and excluded from the study. Then, of nineteen patients, eleven were included in the mass spectrometry analysis because they meet the statistical criteria to be included in the study such as the LFQ value different from zero (LFQ≠0). The IPF patients were then stratified according to the GAP index, a score developed by Ley et al. in 2012 (35) which considers the gender, the age and the physiological lung parameters such as “Diffusion Lung Carbon Monoxide” (DLCO) and Forced Vital Capacity (FVC). The total score obtained from the individual variables allows to classify the patients into 3 stages (Stage I, Stage II and Stage III) which have increasing one-year mortality (**Supplementary Table S1**). The nineteen patients analyzed were either Stage I or Stage II disease with a gap score ranging either from 0-3 or 4-5, while no patients with IPF were diagnosed in Stage III for the impossibility of biopsy diagnosis in patients with very severe pathology and extremely impaired lung function. For the final analysis we selected, according to these characteristics, 3 CTRL patients and 11 IPF patients.

Patients not diagnosed with fibrosing disease, with histologically confirmed non-small cell lung cancer who underwent surgery, were considered as the control population (CTRL patients), since the normal lung parenchyma distal from the peri-tumoral and tumoral area was considered. Then, five Formalin-fixed and paraffin-embedded (FFPE) IPF samples were retrieved from the archive of the Institute of Pathology of the University Hospital of Modena, of which two were excluded from the mass spectrometry analysis for the LFQ value =0 among the technical replicate (**Supplementary Figure 1**).

### 2.3 Deparaffinization and protein extraction for Western blot analysis and preparation of protein samples for mass spectrometry

Replicates of four serial FFPE tissue sections, 10 μm thick, were placed in low-binding Eppendorf tubes and deparaffinized by incubation at room temperature in 1 ml xylene (cod.131769.1611, PanReac-AppliChem, USA) for 10 min. After each incubation, tissue was pelleted at 14.800 rpm for 2 min, and incubation/centrifugation steps were repeated two times. The deparaffinized tissue pellets were then rehydrated by incubation at room temperature in three graded series of 1 ml ethanol (100%, 96%, and 70%, cod.414601, CARLO ERBA, USA) for 10 min. After each incubation, tissue was pelleted at 14.800 rpm for 2 min, and incubation/centrifugation steps were repeated two times. The rehydrated lung tissue sections were resuspended in 0.1 ml extraction buffer made up of 250 mM Tris HCl, pH 9.0, 2%(w/v) SDS, protease inhibitor cocktail (cod. P8340, 1:100, SIGMA, USA) and phosphatase inhibitors (HALT ™ phosphatase Inhibitor Cocktail cod. 78420, 1:100, ThermoScientific, USA). The samples were first incubated on ice for 5 min, mixed by vortexing, then incubated on a heating block at 100°C for 20 min followed by an incubation at 80°C in a Thermomixer for 2 hours with agitation at 750 rpm. After incubation, samples were placed for 1 min at 4°C and then centrifuged for 15 minutes at 14.000 rpm at 4°C. Then, the supernatants containing the extracted proteins were quantified with BCA protein assay kit (cod. 23227, Pierce™ BCA Protein Assay Kit, Thermo Scientific, USA) which was a detergent-compatible formulation (after the dilution 1:10 of samples) and the protein standards were prepared using the same lysis buffer as the samples. Then, 35 µg of total protein lysate for each sample was used for Western Blot. For mass spectrometry analysis, we added 0.4 ml methanol (cod.414816, CARLO ERBA, USA) to 0.1 ml of total protein extraction, previously obtained, 0.1 ml chloroform (cod.415154, CARLO ERBA, USA) and 0.3 ml RNA-ase, DNA-ase, protease free water and centrifuged at 9.000 rpm. After washing the pellet with 1 ml ethanol, the pellet after centrifugation was dissolved in 10 µl of 1% (w/v) Rapigest SF in 25mM NH_4_HCO_3_ (cod. 186001861, Waters, Milford, MA) and 10 µl 50 mM DTT in 25 mM NH_4_HCO_3_. After 1h incubation at 37°C with agitation at 950 rpm, samples were treated with 10 µl of 100 mM iodoacetamide (BioUltra. cod. 144-48-9, Sigma Aldrich, USA) in 25 mM NH_4_HCO_3_, incubated at 37°C for 1 h with agitation at 950 rpm and treated with 90 µl of 25 mM NH_4_HCO_3_. Then, 20 µl of Trypsin-grade (cod. V5111, Promega, WI, USA) was added (0.25 µg/µl) in 25 mM NH_4_HCO_3_ and incubated overnight at 37°C. After about 16 hours, the digestion was stopped by adding 20 µl of 5% (v/v) TFA (Trifluoroacetic Acid cod.102253211, Sigma Aldrich, USA) and incubated at 37°C for 1h with agitation at 950 rpm. The samples were centrifuged at 13.000 rpm for 30 minutes at room temperature and the supernatant containing the tryptic peptide were dried under vacuum using Speedvac evaporators.

### 2.4 LC-MS/MS experimental conditions and data analysis

LC-MS/MS analysis was performed using Q Exactive™ Hybrid Quadrupole-Orbitrap™ Mass Spectrometer (Thermo Fisher Scientific, USA) coupled to a Thermo Ultimate 3000 UHPLC. 11 samples from IPF patients and 3 samples for CTRL patients were analyzed in triplicates by using 50 µg per injection. Chromatographic separation was performed on a Hypersil Gold 2.1x100 mm C18 column (Thermo Scientific, USA). Briefly, an amount of peptide solution to have 50 µg on the column was injected into a Thermo Scientific Dionex Ultimate 3000 UHPLC coupled to a Thermo Ultrahigh-resolution Q Exactive mass spectrometer (Thermo Scientific, Bremen, Germany) and detailed information is provided as **Supplementary Material S1**. Raw MS files were analyzed with the MaxQuant software version (v1.6.2.6) (Max Planck Institute, Martinsried, Germany) (36) against the Human Uniprot database (NCBI: txid9606, 2023_01, 245,871,724 sequence entries), including both the protein modifications such as carbamidomethylation (C) (fixed), oxidation (M) (variable) and N-terminal acetylation (variable) and the enzyme specificity set to trypsin; the maximum missed cleavages were set to 2 and the parent peptide masses searched with a maximal initial mass deviation of 10 p.p.m. The false discovery rate (FDR) filtration on the peptide spectrum was set to 0.01. Proteins were quantified using LFQ (Label-Free Quantification) intensity. Thus, proteins with LFQ ≠ 0 in all three replicates for each sample were subjected to bioinformatics analysis using the freely available software Perseus v1.6.15.0 (Max Planck Institute, Martinsried, Germany, www.perseus-framework.org) (37). Here, LFQ intensities were log_2_ transformed and there were retained only samples with normal data distribution. Results were then filtered to remove contaminants, reverse matches, proteins only identified by site and a MS/MS spectral count ≥2. Subsequently, data were row filtered according to valid values (minimum valid percentage, 75%) and then normalized by median subtraction. Then, a downward shift of 1.8, and a width of 0.3 standard deviations was set in the normal distribution for missing values. For the pairwise comparisons performed with the Volcano plot, the X-axis showed the fold change calculated by the difference between the average of log_2_ values (Δlog_2_ (LFQ intensity)) of proteins detected in CTRL patients *vs*. proteins detected in stratified IPF patients. The related statistical analysis was performed with Student’s t-test for two-tailed unpaired data where the p-values were adjusted using the FDR-based permutation method (FDR: 0.01). Thus, the fold change FC ≥ 0.5 and ≤0.5 with a consistent p-value of 0.05 represented a cutoff for proteins differentially expressed.

### 2.5 Bioinformatics analysis and enrichment pathways

A 3-way Venn diagram was built to show the number of unique and shared detected proteins in CTRL patients, Stage I and Stage II patients by using the interactiVenn Web application (http://www.interactivenn.net/) (38). Functional enrichment analysis was performed with FunRich software which is an open-access, standalone functional enrichment and network analysis tool (www.funrich.org) (39). Then, biological process, biological pathway, molecular function, and cellular component represented as donuts charts, showed the functional enrichment as a percentage differentially expressed between CTRL and IPF patients, (significant when p-values were <0.05). Then, to identify the molecular interactors of each differentially expressed protein, the search tool for retrieval interacting genes/proteins v11.5 (STRING) was used (https://string-db.org/) (40). Finally, Network Analyst (41) was used to identify the potential interaction among the differentially expressed proteins allowing the construction, visualization and the analysis of complex networks through the KEGG pathway. Here, the protein lists with differentially expressed proteins were uploaded to perform protein-protein interaction analysis either based on STRING analysis or comprehensive literature and built a second- order network.

### 2.6 Western blot and immunohistochemical analysis

Western blot (WB) analysis was performed according to standard protocols. Briefly, 35 µg of total proteins isolated from FFPE tissue blocks, as previously described, were separated by SDS-PAGE gel electrophoresis. Then, proteins were transferred to a nitrocellolose membrane, and primary antibodies Anti-COL1A1 (E6A8E) (1:1000, cod. 39952, Cell Signaling; MA, USA), Anti-pSmad2/3 (D7G7) (1:1000, cod. 8685, Cell Signaling; MA, USA) Anti-β-Actin-Peroxidase antibody (1:30.000, cod. A3854, Sigma-Aldrich, USA), Anti-LCP1 (LCP1, 1:1000, cod. Ab236104, abcam, Cambridge, UK) Anti-Peroxiredoxin 2/PRP (PRDX2, 1:1000, cod. Ab109367, abcam, Cambridge, UK), Anti-Lumican (LUM, 1:1000, cod. Ab168348, abcam, Cambridge, UK), Anti-mimecan (OGN, 1:1000, cod. PA5112635, ThermoScientific, USA) and Anti-TAGLN2 (1:1000, cod. PA551664, ThermoScientific, USA) were incubated overnight at 4°C. After the incubation of the membranes for 1h at room temperature with horseradish peroxidase-conjugated anti-rabbit secondary antibodies (1:30.000; cod. A120-101P, Bethyl, MA, USA) followed by the incubation with the ECL (Pierce ECL, cod.32209, Thermo Scientific, USA), specific proteins bands were revealed. The corresponding IPF samples and CTRLs were then cut to obtain 5 μm sections and used to perform IHC analysis where the antibodies mentioned above were employed according to the working dilution for IHC (LCP1, 1:400, LUM, 1:100, OGN, 1:100, PRDX2, 1:500, TAGLN2, 1:300). The IHC reaction was performed using the DAB Ultraview Universal Detection Kit and the BenchMark XT fully automated IHC slide staining instrument (Roche, Basel, CH).

### 2.7 Statistical Analysis for western blot quantification

Ordinary One-Way ANOVA was used to determine the statistical significance of the intensity band of Western Blot from the quantification, both for single patients and for all patients stratified in three groups (CTRL, Stage I, and Stage II IPF patients).

## 3 Results

### 3.1 Total protein isolation from FFPE lung tissue and mass spectrometry analysis in stratified patients

**Figure 1:**
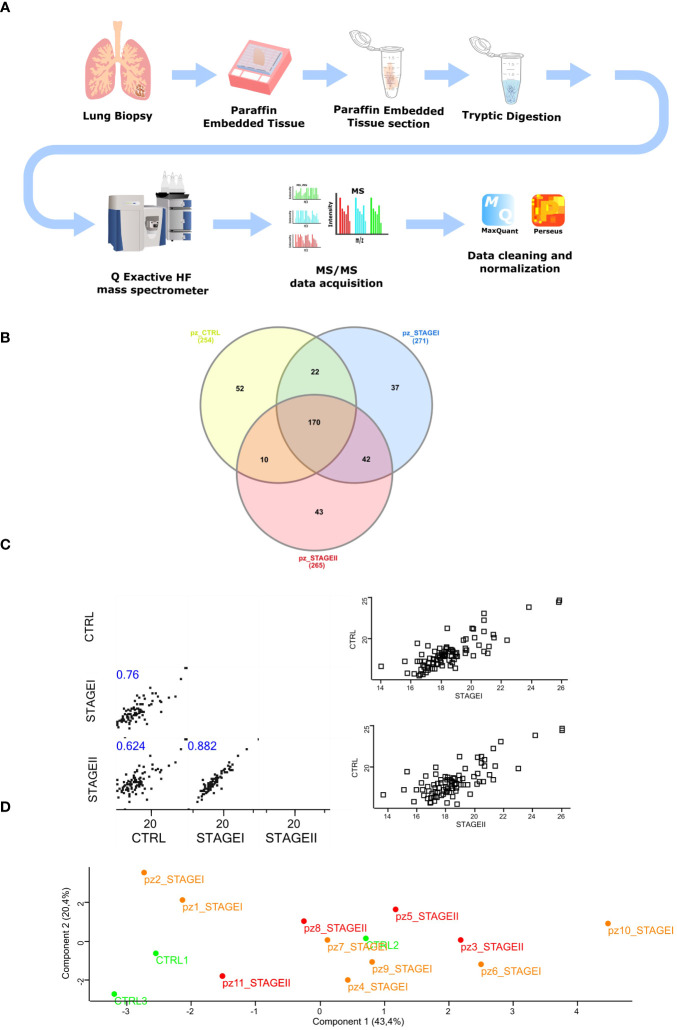
Isolation of total protein and mass spectrometry analysis of FFPE IPF lung tissue **(A)** The experimental workflow followed in our study started with FFPE samples from IPF lung biopsy cutting in 10 µm tick sections. Samples were then subjected to tryptic digestion, processed with Q Exactive™ Mass Spectrometer coupled to a UHPLC and analyzed with MaxQuant software followed by Perseus Analysis. **(B)** To identify those proteins specific for IPF patients, the Venn diagram was performed submitting the proteome lists of CTRL, STAGEI, STAGEII patients, which account for 254, 271 and 265 proteins, respectively. As the Venn diagram shows, 37 proteins (13.65%) were exclusive to STAGEI patients while 43 proteins (16.22%) were exclusively to STAGEII patients. Common proteins among three groups were 170, while common proteins exclusively shared between CTRL and STAGEI were 22, while common proteins exclusively shared between CTRL and STAGEII were 10, showing a more similar protein profile between CTRL and STAGEI rather than STAGEII. **(C)** Multi-scatter plot of averaged profiles among the three groups represents the disease progression giving the strong similarities in the subsequent stages of IPF, where CTRL lung tissue samples are more similar to STAGEI patients than to STAGEII (Spearman rank correlation coefficient 0.759 *vs* 0.624, p-value<0.05), while STAGEI lung tissue are more similar to STAGEII (Spearman rank correlation coefficient = 0.882, p-value<0.05). **(D)** Principal component analysis (PCA) shows difference between two CTRL patients (CTRL1, CTRL3, green dots) and the majority of IPF patients at Stage I and Stage II (orange and red dots, respectively) with the exception for patient 1 and patient 2 at Stage I (Component 1, 43.4%), while STAGEI and STAGEII have a very similar proteomic profile.

The experimental workflow followed for the molecular profiling based on the proteomic analysis of both IPF and control lung tissues is shown in **Figure 1A** (**Figure 1A**) and discussed in detail in Materials and Methods section. Briefly, a total of 11 archival FFPE from surgical lung biopsy (SLB) of patients with a proven diagnosis of IPF (Ps IPF) and 3 archival FFPE of patients that underwent SLB for lung cancer disease referred to CTRL patient’s population (Ps CTRL) were obtained from Pathology Department (**Table S2**, **Supplementary Figure 1**). To maximize the protein yield from FFPE tissue blocks for LC-MS/MS analysis, we firstly established an experimental approach to isolate the total proteins based on a previously described protocol (42). Then, isolated proteins were subjected to tryptic digestion and peptides were then measured on Q Exactive™ Hybrid Quadrupole-Orbitrap™ Mass Spectrometer followed by data analysis in MaxQuant (36). On average, our protocol yielded >400 hundred total proteins in each sample that were technically above the total proteins identified from fresh-frozen tissues (43), probably due to the effect of formalin fixation that might cause formalin-induced crosslinking (44) (45)and paraffin embedding in terms of peptide extraction efficiency (see Study Limitations). Then, we used the LFQ intensity to determine the relative abundance of total proteins in each sample and selected only the samples whose technical replicates had adequate correlation coefficients through Perseus Software(37) (96%<R^2^<98%) (**Supplementary Figure 1**). Then, the LC-MS/MS spectral counts that were considered consistent when LFQ intensity was different from zero in all technical replicates, gave us a total of 254 proteins for CTRL patients, 271 proteins for Stage I patients, and 265 proteins for Stage II patients as shown in Venn diagrams performed for three sets analysis. Of these, 170 proteins were common in all three groups, while CTRL and Stage I patients shared 22 proteins, CTRL and Stage II patients shared 10 proteins and Stage I and Stage II patients shared 42 proteins (**Figure 1B**). Subsequently, the 170 common proteins in the three patient groups were filtered for valid values (minimum percentage value 70) and the resulting 87 differentially expressed common proteins were used to perform the multi-scatter plot representing the non-linear correlation of averaged profiles among the CTRL, Stage I and Stage II patients. Here, the analysis of the three groups of patients, clearly revealed high similarity between subsequent stages, where CTRL patients were more similar to Stage I patients compared to Stage II (II (Spearman correlation coefficient: 0.759 *vs* 0.624) representing the disease progression, while Stage I and Stage II patients showed the highest similarity (Spearman correlation coefficient=0.882) (**Figure 1C**). In addition, the principal component analysis (PCA) revealed a good separation of two CTRL patients out of three (CTRL1, CTRL3 green dots) with respect to IPF patients (Stage I and Stage II, orange and red dots respectively), while Stage I and Stage II patients subtype clustered almost together, also revealing that Stage I and Stage II patients (Component 3, 11.8%) share similar proteomic profile distribution (**Figure 1D**).

### 3.2 Pathway enrichment analysis of differentially expressed proteins

**Figure 2:**
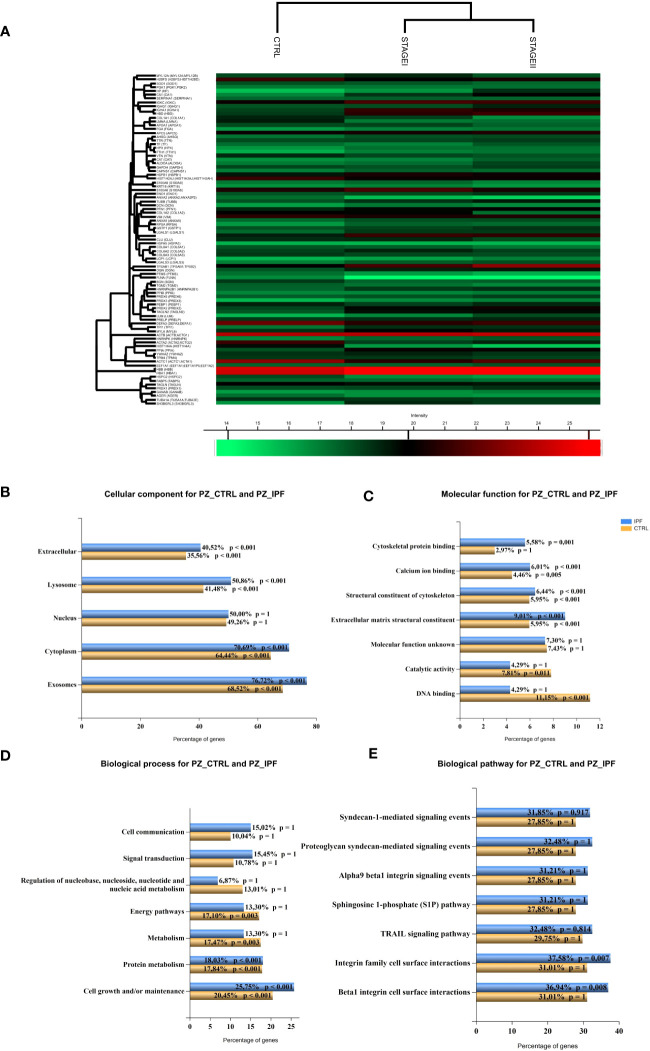
Pathway enrichment analysis of differentially expressed proteins between CTRL and IPF patients **(A)** Hierarchical clustering of protein intensities was log_2_ transformed, Z-scored and normalized between groups with Hierarchical Clustering of Perseus Software resulting in the heat map showing the protein names of differentially expressed proteins in the left column. High and low expression are shown in the heat map in red and green, respectively. Different clusters of protein groups are shown in the dendrogram. KEGG Pathway enrichment analysis of differentially expressed proteins between CTRL and IPF patients (StageI and StageII) shows **(B)** the cellular component **(C)** the molecular function, **(D)** the biological process and **(E)** the biological pathway represented as bar charts where percentage of represented pathways of CTRL and IPF patients were shown. (p value <0.05, p value <0.001).

To detect distinct protein-based subtypes of disease state, we performed hierarchical clustering based on the data matrix to generate an output matrix or heatmap resulted in high and low expression of proteins shown in red and green, respectively, for both the CTRL patients and Stage I and Stage II patients (**Figure 2A**). Then, the functional analysis together with the corresponding enrichment factor and p-value enabled the delineation of multiple categories of differentially expressed proteins enriched in the three groups of patients that were: the ECM receptor interaction, protein-DNA complex, nucleosome, the hemoglobin complex, the focal adhesion and protein digestion and absorption (**Supplementary Table S3**). To put a special emphasis on differentially expressed proteins between CTRL and IPF patients, we implemented the functional enrichment analysis with FunRich software tool (39) (46) (47), showing the cellular component (**Figure 2B**), the molecular function (**Figure 2C**), the biological process (**Figure 2D**) and the biological pathway (**Figure 2E**), to which the differentially expressed proteins belong. In particular, the enrichment analysis revealed that the cellular component associated with IPF were mainly represented by the exosomes (76.7% IPF patients *vs* 68.5% CTRL), the extracellular components (40.5% IPF patients *vs* 35.6% CTRL), and lysosome (50.9% IPF patients *vs* 41. 5% CTRL) (**Figure 2B**), reflecting in part the molecular function (**Figure 2C**) with the extracellular matrix structural constituent most represented in IPF patients (9% IPF patients *vs* 5.9% CTRL) as well as the cytoskeletal protein binding (5.6% IPF patients *vs* 3% CTRL). In addition, the biological process (**Figure 2D**) of differentially expressed proteins revealed that only the cell growth and maintenance (25.8% IPF patients *vs* 20.45% CTRL) showed a statistical significance, while the signal transduction (15.5% IPF patients *vs* 10.7% CTRL, ns), and the cell communication (15% IPF patients *vs* 10.04% CTRL, ns), were slightly enriched in IPF patients compared to CTRL. Finally, we did not observe statistical significance of gene enrichment in the biological pathways of IPF disease involving the TGF-β receptor signaling, the regulation of cytoplasmic and nuclear SMAD2/3 signaling, and β1 integrin cell surface interactions (**Figure 2E**). Finally, given the limitation of the total proteins detection, the functional enrichment analysis partially recapitulate the cellular and molecular changes in fibrotic tissue as the aberrant deposition of extracellular matrix and the cytoskeletal distortion involved in IPF development (48, 49).

### 3.3 Identification of differentially expressed proteins between CTRL and IPF stratified patients

**Figure 3:**
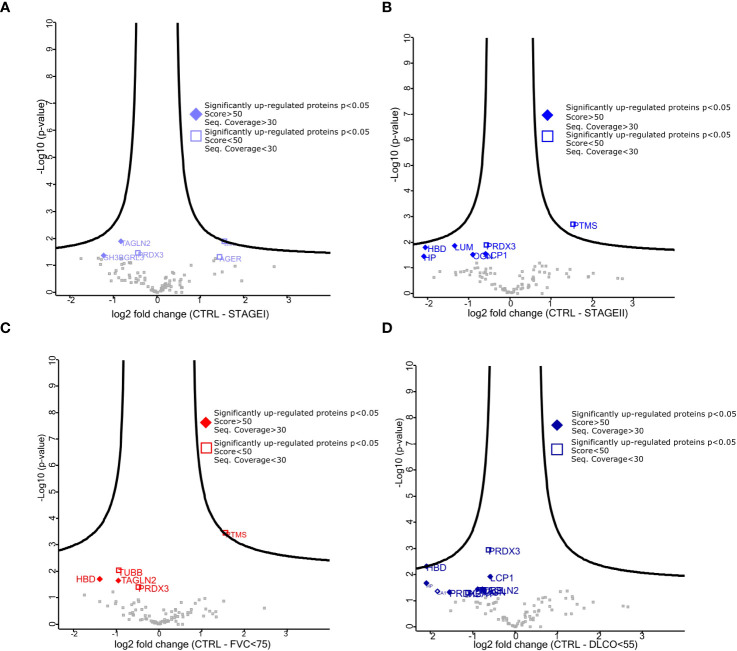
Differentially expressed proteins among stratified IPF patients. **(A–C)** Volcano plot showed both up-regulated (left) and down-regulated (right) proteins in all pairwise comparisons. Volcano plots show log 2 FC (x-axis) and −log10 value of p-value (y-axis). The thresholds are set for a base log 2 > 0.5 and FDR p value > 0.05. **(A)** represents the pairwise comparison between CTRL and StageI patients, **(B)** represents the pairwise comparison between CTRL and StageII patients, **(C)** represents the pairwise comparison between CTRL and patients having FVC<75, **(D)** represents the pairwise comparison between CTRL and patients having DLCo<55. The quality of mass spectrometry analysis (fill diamond proteins) was assessed for proteins showing sequence coverage >30 and score value >80.

**Figure 4:**
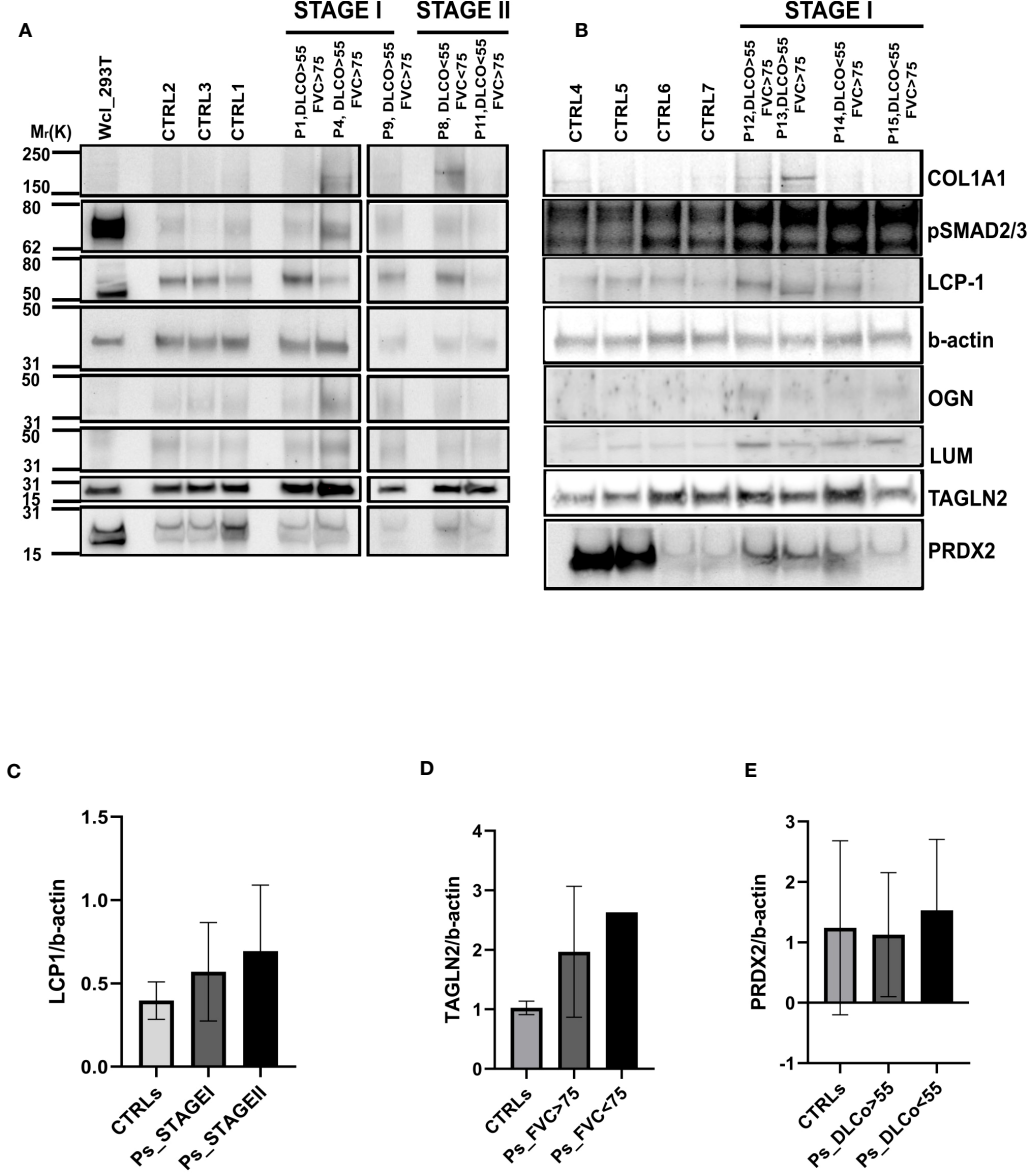
Validation of differentially expressed proteins through Western Blot Analysis **(A)** Western blot of the five differentially expressed proteins: LUM, OGN, LCP1, PRDX2 and TAGLN2 in three CTRL patients (CTRL 2, 1,3), three Stage I patients (p1, p4, p9) and two Stage II patients (p8, p11). COL1A1 and pSMAD2/3 were used to demonstrate their up-regulation in the IPF patients compared to CTRL patients as shown in p1, p4, p9 and to a lesser extent in patient 8. **(B)** Western blot of the five differentially expressed proteins: LUM, OGN, LCP1, PRDX2 and TAGLN2 in four CTRL patients (CTRL4, 5, 6, 7) and four Stage I patients (p12, p13, p14, p15).The bar charts representing the densitometric analysis of western blot show: **(C)** LCP1 intensity value normalized against b-actin both for the three main groups of patients represented by CTRL, STAGEI and STAGEII, **(D)** TAGLN2 intensity value normalized against b-actin for the three groups of patients represented by CTRL, patients having DLCO>55 and patients having DLCO<55 and **(E)** PRDX2 intensity value normalized against b-actin both for the three groups of patients represented by CTRL, patients having FVC>75 and patients having FVC<75. Data are mean ± s.d. deriving from three different western blots, ordinary, one-way Anova, not significant.

Given the differences between CTRL and IPF patients shown in the pathway enrichment analysis and functional ontology classification, we first aimed to detect differentially expressed proteins between CTRL patients and Stage I**/**Stage II IPF patients. Thus, the Volcano Plots, showing the log_2_ values of protein Fold Change in terms of LFQ intensity values (log_2_ fold changes), revealed two up-regulated proteins that characterized Stage I patients compared to CTRL patients that were transgelin 2 (TAGLN2) and SH3 Domain-Binding Glutamic Acid-Rich-Like Protein 1 (SH3BGLR3) (**Figure 3A**). Additionally, the up-regulated proteins for Stage II patients compared to CTRL patients were lumican (LUM), mimecan (OGN), and lymphocytes cytosolic protein1 (LCP1) (**Figure 3B**). We subsequently confirmed our observation on the high similarity of proteomic profile of Stage I and Stage II patients in The Volcano Plot showing there are no significant differentially expressed proteins that meet the criteria in terms of score and sequence coverage between stages, to be considered for further analysis (**Supplementary Figure 3**). Then, we thought to compare CTRL patients with IPF patients who had a decline in respiratory function to identify proteins predictive of lung impairment. Specifically, we compared CTRL patients with IPF patients having FVC <75 (cut-off from Stage I to Stage II disease) (**Figure 3C**) and CTRL patients with IPF patients having DLCO<55 (cut-off from Stage I to Stage II disease) (**Figure 3D**). As shown in the specific Volcano Plots, these two further comparisons confirmed some proteins up-regulated in Stage II patients compared to CTRL such as LCP1 and OGN up-regulated in IPF patients having DLCO<55 (**Figure 3D**). In addition, PRDX2 was uniquely significantly up-regulated in IPF patients having DLCO<55, while TAGLN2 was up-regulated in patients characterized by both FVC<75 and DLCO<55. To assess the presence of proteins that could be indicative of the lung function decline and therefore of the disease severity, we thought to focus on the up-regulated proteins in patients with IPF Stage II or with compromised lung function (e.g FVC<75, DLCO<55), not further investigated, in the present study, the differentially and uniquely expressed proteins in Stage I patients (e.g SH3BGRL3, **Figure 3A**) as well as patients having FVC>75 and DLCO >55 (data not shown). Then, we performed western blot analysis both on FFPE samples from five IPF and three CTRL patients used for MS (**Figure 4A**) and other new FFPE samples from CTRL patients compared with other FFPE samples from new patients with IPF (**Figure 4B**),with specific antibodies for the selected up-regulated proteins: LCP1, LUM, OGN, PRDX2, and TAGLN2. The western blots shown in **Figures 4A, B** with the relative quantification of signal intensity for each protein normalized on β-actin, confirmed an up-regulation trend of both LCP1 for Stage II patients (**Figures 4A, C**), and to a lesser extent PRDX2 (**Figures 4A, E**) for Stage II patients having DLCO<55 compared to CTRLs, while the TAGLN2 up-regulation was confirmed for a single patient (p8 Stage II) having the FVC<75 and Stage I patients compared to CTRL patients (**Figures 4A, B, D**). Then, the low undefined signal intensity detected from western blot analysis for OGN and LUM (**Figure 4A**), did not give us the possibility to confirm the up-regulation of these protein from mass spectrometry in IPF patients Stage II, through western blot quantification. Finally, we performed further experimental validations based on immunohistochemical (IHC) analysis (**Supplementary Figure 4**). The IHC images showed that the signal intensity associated to TAGLN2, to a lesser extent to PRDX2, LCP-1, LUM and OGN proteins was higher in the lung tissue of both patients (Patient 1 and Patient 11) compared to CTRLs (**Supplementary Figures 4A–E**). In particular, LCP-1 (**Supplementary Figure 4C**) and OGN (**Supplementary Figure 4E**) were highly expressed in Stage II patient compared to Stage I patient retracing the results of Mass Spectrometry analysis considering the patients stratification.

### 3.4 Molecular interactors and functional network of significantly up-regulated proteins in patients with severe IPF

**Figure 5:**
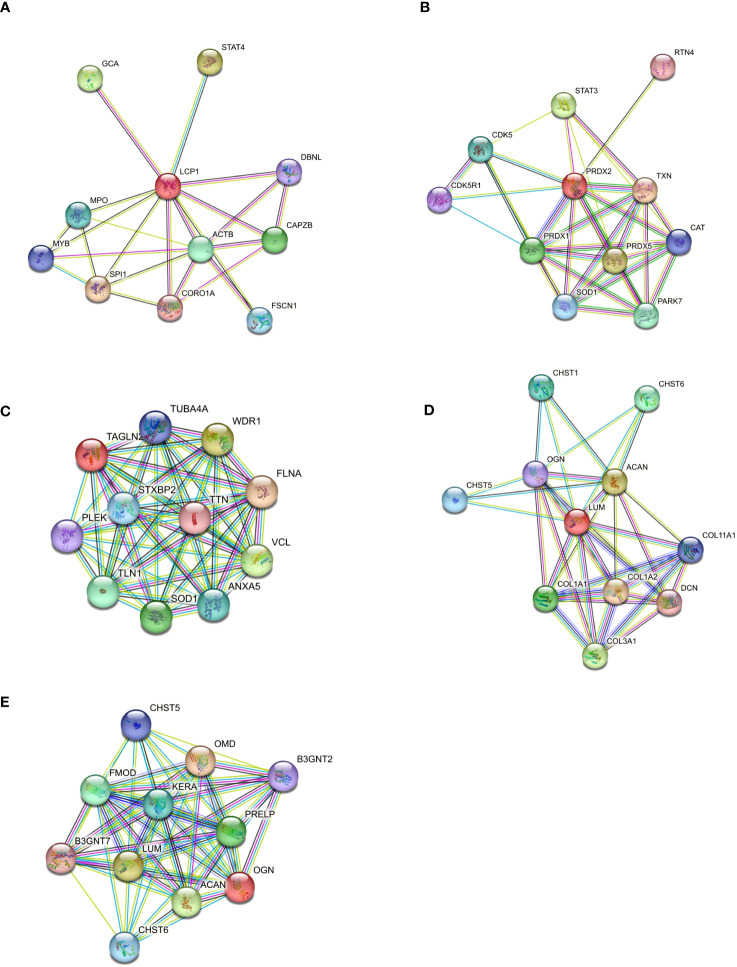
Protein–protein interaction of differentially expressed proteins. **(A)** Differentially expressed proteins were used to build a molecular network using STRING software to analyze the molecular partners for each selected protein **(A–E).** The molecular interactors source, is based on Text Extraction, Experiments, Database, Coexpression, Neighborhood, Gene Fusion, and Co-occurrence.

**Figure 6:**
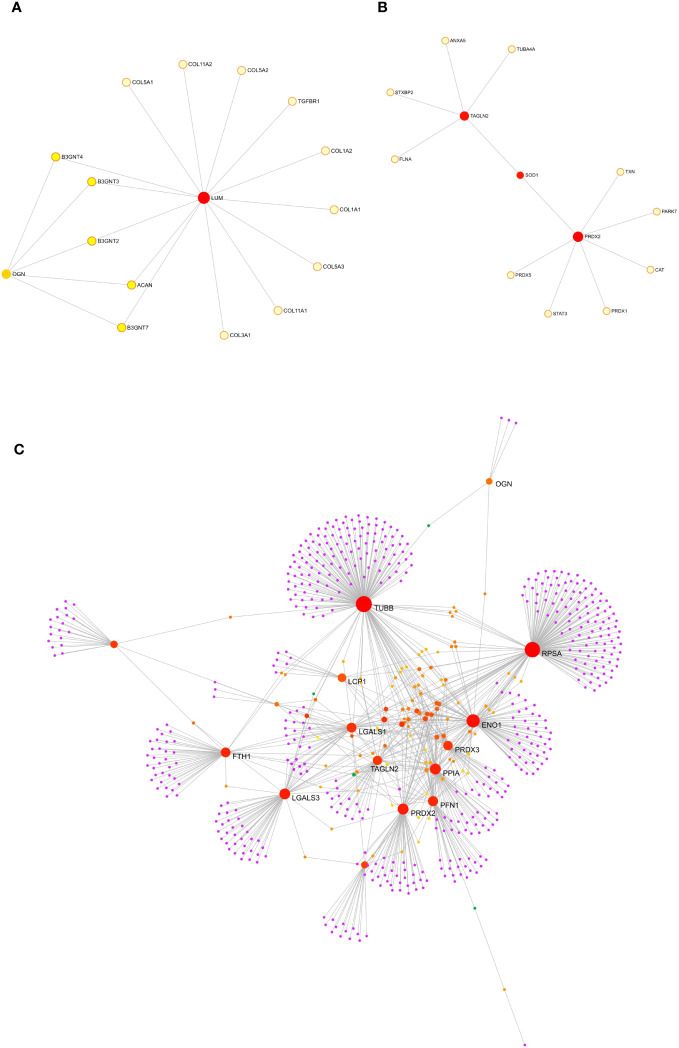
Protein-protein interaction analysis though GeneNetwork Analyst. Protein-protein interaction (PPI) networks was built with GeneNetwork Analyst software. The interaction network based on STRING interactome database by using the five differentially expressed proteins resulted in one interaction sub-networks **(A)** containing 16 nodes and 19 edges showing the interaction between LUM and OGN. The second subnetwork **(B)** made up of 13 nodes and 12 edges showed the interaction between PRDX2 and TAGLN2. **(C)** By adding in the analysis 20 other proteins that were not statistically significant but differentially expressed between CTRL and Stage II patients a second order network was performed based on the literature curated comprehensive data that showed one complex sub-network of about 643 nodes and 870 edges were LCP1, PRDX2, OGN and TAGLN2 linked together with other molecular interactors.

To gain insight into the role of the five statistically up-regulated proteins (LCP1, PRDX2, TAGLN2, LUM, OGN), in IPF patients at Stage II and/or with impaired lung function, we explored their known and predicted molecular interactors using the bioinformatic software STRING v11.5 (40). Specifically, LCP1 (**Figure 5A**), which is an actin-binding protein showed the highest score with Transcription factor PU.1 (SPI1, score: 0.916), Signal transducer and activator of transcription 4 (STAT4, score: 0.904) and Grancalcin (GCA, score: 0.855). For PRDX2 (**Figure 5B**), implicated in the reduction of hydrogen peroxide, the three highest score predicted functional partners, were Thioredoxin (TXN, score: 0.997), Peroxiredoxin 5 (PRDX5: 0.983), Signal transducer and activator of transcription 3 (STAT3, score: 0.974), while for TAGLN2 (**Figure 5C**) which is involved in the regulation of cell morphology, the three highest score can be attributed to Filamin-A (FLNA, score: 0.948), WD repeat-containing protein 1 (WDR1, score: 0.931) and Vinculin (VCL, score: 0.930). LUM (**Figure 5D**) which may regulate collagen fibril organization and the epithelial cell migration, showed the highest score predicted functional partners Collagen alpha-2(I) chain (COL1A2, score: 0.996), Aggrecan core protein (ACAN, score: 0.983) and Collagen alpha-1(III) chain (COL3A1, score: 0.982). Finally, OGN (**Figure 5E**) which is involved in ectopic bone formation, and regulating the osteoblast differentiation, presented as the three highest score functional partners Osteomodulin (OMD, score: 0.964), LUM (score: 0.943) and ACAN (score: 0.940). Thus, we found that LCP1 and PRDX2 are brought together by the molecular interaction with two STAT family members (STAT4 and STAT3, respectively), while the TAGLN2 shared with PRDX2 the Superoxide Dismutase 1 protein (SOD1), despite not being part of the first-ranked molecular interactors. Moreover, LUM and OGN which remained “unassociated” from the previous proteins were predicted molecular partners whose interaction was experimentally determined. Finally, we might speculate the presence of two networks: the first one made up of LCP1, PRDX2 and TAGLN2 and the second one made up of LUM and OGN that might reveal key molecular connected interactions that might impact the IPF progression. Furthermore, we implemented the bioinformatic analysis with the Gene Analyst software (41) in the context of protein-protein interaction (PPI) networks, where nodes represent functions and edges are determined by the overlap ratio between genes associated with the two functions. Thus, we built an interaction network based on the STRING interactome database by using the five differentially expressed proteins that resulted in 2 interaction sub-networks. The first subnetwork (**Figure 6A**) containing 16 nodes and 19 edges showed the interaction between LUM and OGN as well as a mild increase in multiple components of the protein digestion and absorption (COL5A1, COL5A2, COL11A2, COL1A2, COL1A1, COL5A3, COL3A1), the ECM-receptor interaction and focal adhesion (COL1A2, COL1A1) and glycosphingolipid biosynthesis (B3GNT4, 3, 2). The second subnetwork (**Figure 6B**) containing 13 nodes and 12 edges showed the interaction between PRDX2 and TAGLN2 as well as a mild increase in multiple components of the peroxisome (SOD1, CAT, PRDX5, PRDX1) and proteoglycans in cancer (FLNA, STAT3). We subsequently expanded the network by adding in the analysis 20 other proteins that were not statistically significant but differentially expressed between CTRL and Stage II patients, to better contextualize the role of LUM, OGN, PRDX2, TAGLN2, and LCP1 in the disease progression. To do this, we performed a second-order network based on the literature-curated comprehensive data that showed one complex sub-network of about 643 nodes and 870 edges (**Figure 6C**). Here, LCP1, PRDX2, OGN and, TAGLN2 together with other molecular interactors, linked with highest hits, according to KEGG database, to pathways in cancer, PI3K-AKT and MAPK signaling pathways (**Supplementary Figure 5**). The other sub-network showed the unassociated LUM protein (**Supplementary Figure 6**). Thus, our results based on functional network analysis and thus a predictive bioinformatic model could indicate that PRDX2, LCP1, OGN and TAGLN2 might impact the IPF progression through mechanisms common to cancer.

## 4 Discussion

Previous study have tested the applicability of different experimental approaches on FFPE samples that represent a valuable resource to study the molecular signature of IPF patients when there is no availability of fresh-frozen material (33, 50). We decided to use quantitative proteomics through LC-MS/MS to retrospectively examine the presence of differentially expressed proteins in the lung tissue of IPF patients compared to controls. Human CTRL tissues were represented by SLB of lung cancer patients whose normal parenchyma was distal to non-small-cell lung cancer (NSCLC) (50). The analysis of LC-MS/MS of both IPF and CTRL FFPE samples resulted in the detection of 400 proteins on average, with sufficient sequence coverage, comparable to those obtained from mass spectrometry analysis performed on FFPE samples in other diseases (51, 52).

First, our study demonstrates that the proteomic profiling of FFPE samples from lung biopsies by quantitative proteomic is feasible although not optimal if the conditions of formalin fixation and tissue storage are not appropriate; second, and importantly, label-free quantitative proteomics allowed us to identify differentially expressed proteins between the control patients and the various patients’ groups (FDR-adjusted p < 0.05) based on both the stages of IPF (Stage I and Stage II) and lung pathophysiological parameters (DLCO<55, FVC<75), where 55 for DLCO and 75 for FVC were considered cut-off values that mark the stage transition of IPF. Five total proteins were differentially expressed among different pairwise comparisons such as CTRL *vs*. Stage I, CTRL *vs*. Stage II, CTRL *vs*. FVC<75 and CTRL *vs*. DLCO<55. In particular, LUM was exclusively expressed in IPF patients at Stage II, while LCP1 and OGN were expressed in Stage II patients and patients having DLCO<55. Moreover, TAGLN2 was exclusively associated with patients having FVC<75, while PRDX2 was exclusively associated to patients having DLCO<55.

Lumican (LUM) and mimecan (OGN), which were exclusively related to Stage II IPF patients, are small leucine-rich proteoglycan (SLRP) families of proteins that represent key components of the ECM. In particular, Lumican, is expressed in the ECM of many tissues such as skin, kidney, breast, colon, pancreas, and cartilage (53). Furthermore, it has been found that LUM is upregulated in acute lung injury related to mechanical ventilation because of high tidal volume that induces epithelial-mesenchymal transition (EMT) through the activation of the extracellular signal-regulated kinase 1/2 (ERK 1/2) pathway (54). Concerning pulmonary fibrosis, it has been shown an upregulation of LUM in the fibrotic lesions of patients with advanced disease, that promotes human monocyte differentiation into fibrocytes (55). Recently, it has been demonstrated that lumican expression levels were increased in early human and experimental ARDS and linked to disease severity. Here, Lumican induces both the transdifferentiation of lung fibroblasts into myofibroblasts and the epithelial-mesenchymal transition through the ERK pathway (56). Moreover, LUM upregulation has been demonstrated in some cancers such as breast carcinoma, related to increased levels of metastasis, melanoma (53, 57) and in cancer associated fibroblasts (CAFs) of esophageal squamous cell carcinoma (ESCC) (58). OGN has been recently related to interstitial lung disease where it has been demonstrated that the ectopic expression of miR-140 and the subsequent down-regulation of OGN in bleomycin-treated mice lung fibroblasts, resulted in increased lung fibroblast apoptosis and Wnt3a expression, together with reduced proliferation and pulmonary fibrosis (59). Moreover, several studies describe the impact of OGN on fibrosis in other organs. Recently, a study based on proteomic analysis, demonstrated an increased expression of OGN in atherosclerotic plaques at the stage of fibrosis and calcification (60). In fibrotic renal disease, OGN may increase and accumulate in areas of tubulointerstitial fibrosis (61). Increased OGN levels have been shown in progressive myelofibrosis which plays a key role in Duchenne muscular dystrophy (62). Moreover, it has been shown that OGN can either exhibit protumorigenic or antitumorigenic functions. In some cancers OGN is down-regulated compared with normal tissues, as described in squamous cervical (63) cancer gastric cancer (64) colorectal cancer (65), vaginal cancer (63), invasive ductal breast carcinoma (66), laryngeal carcinoma (26), and thyroid tumors, while Zheng and colleagues demonstrated different expression of OGN as a marker for differential diagnosis between non-small-cell lung cancers (positive for OGN) and small-cell lung cancers (negative for OGN) (67). LCP1 or l-plastin is an actin binding that was up-regulated in Stage II IPF patients and in patients having DLCo<55. LCP-1 has never been investigated in interstitial diseases, but it is up-regulated in the serum of patients with Nonalcoholic fatty liver disease (NAFLD) that may lead to the development of liver cirrhosis and fibrosis. (68). Indeed, LCP-1 has been identified as a biomarker of progression in several malignant tumors such as oral squamous cell carcinomas (OSCCs) (69), colon cancer (70), (71), correlating positively with advanced tumoral stages. TAGLN2 which is an actin-binding protein that modulates the actin cytoskeleton dynamics was statistically up-regulated in patients with lung impairment having FVC<75 compared to CTRL patients. TAGLN2 was identified as a biomarker in the development of pulmonary fibrosis since it triggered the activation of the TGF-beta/Smad3-pathway (72). Moreover, several studies stated that TAGLN2 can modulate multiple cancer-related processes, including cell migration, proliferation, differentiation, and apoptosis in glioma and gastric cancer (73), (74). Finally, PRDX2 which encodes a member of the peroxiredoxin family of antioxidant enzymes, was found up-regulated among 30 proteins in fibrotic kidney fibroblasts (TK188) compared to normal kidney fibroblast (TK173), suggesting a role in the progression of renal fibrosis (75). Moreover, PRDX2 promotes both the proliferation of colorectal cancer increasing the ubiquitinated degradation of p53 (76) and the proliferation and metastasis of Non-Small cell lung cancer (77). Thus, it is important to point out that the up-regulated proteins that we identified have been already detected in multi-organ fibrosis and they have been already characterized as biomarkers of cancer progression highlighting the similarity between fibrogenesis and carcinogenesis where the myofibroblasts and the Cancer Associated Fibroblast (CAFs) respectively, play a pivotal role.

## 5 Study limitations

The most significant limitation of our study was the low yield from our FFPE samples, in terms of the total isolated proteins. This was the result of the suboptimal quality of our archived analyzed samples that were the best available, considering the paucity of patients with this rare condition, without other concomitant diseases and, a triggering cause (e.g., secondary pulmonary fibrosis). Despite these trouble shootings, we conducted the mass spectrometry analyses trying to be as stringent as possible with statistics (see Material and Method) considering only the samples with the best protein distribution (Max Quant) and the highest Pearson’s correlation coefficient among the technical triplicate for each patient (Perseus). Finally, we were not able to analyze, for each patient, a specific fibrotic region histologically characterized by fibroblastic foci, that might be more representative of the proteins involved in IPF progression. In the future, we wish to overcome the limitations of MS analysis of FFPE samples using fresh/frozen tissues and the Nano-UHPLC for a better detection sensitivity.

## 6 Conclusions

With our study we have demonstrated the feasibility of LFQ analysis from FFPE IPF lung samples, although their suboptimal quality that is responsible for the low protein detection in MS compared to a fresh-frozen tissue. Despite the low protein counts might lead, as in our case, to having an incomplete molecular profile of idiopathic pulmonary fibrosis patients, not withstanding, our findings will favor the use of FFPE samples for new studies using the described protocol or an implementation of it. The five differentially expressed proteins in advanced IPF patients, similar to those already found for potential cancer proliferation, might identify new potential biomarkers of disease progression, supporting the existence of common molecular mechanisms to both pathologies which need to be further studied.

## Data availability statement

The datasets presented in this study can be found in online repositories. The name of the repository/repositories and accession number(s) can be found in the article/supplementary material (https://figshare.com/articles/dataset/Raw_mass_spectrometry_data_Samarelli_and_Tonelli/23995500).

## Ethics statement

The studies involving humans were approved by University of Modena and Reggio Emilia (557/2019/SPER/AOUMO). The studies were conducted in accordance with the local legislation and institutional requirements. The participants provided their written informed consent to participate in this study. The animal study was approved by University of Modena and Reggio Emilia (557/2019/SPER/AOUMO). The study was conducted in accordance with the local legislation and institutional requirements.

## Author contributions

AVS: Conceptualization, Data curation, Investigation, Methodology, Resources, Supervision, Validation, Writing – original draft. RT: Conceptualization, Data curation, Writing – original draft. GR: Writing – original draft, Data curation, Investigation, Methodology, Validation. GB: Conceptualization, Writing – original draft. DA: Conceptualization, Data curation, Writing – original draft. FGo: Conceptualization, Data curation, Writing – original draft. AM: Conceptualization, Data curation, Writing – original draft. MC: Writing – original draft, Data curation, Investigation, Validation. LF: Writing – original draft, Investigation, Methodology. FGe: Writing – original draft, Data curation, Investigation, Methodology. DP: Investigation, Methodology, Writing – original draft. LM: Investigation, Writing – original draft. IC: Investigation, Writing – original draft. VM: Data curation, Methodology, Writing – original draft. BA: Data curation, Investigation, Writing – original draft. LT: Data curation, Investigation, Writing – review & editing. SR: Writing – original draft, Data curation, Investigation. SB: Writing – original draft, Data curation, Investigation, Methodology. SM: Writing – original draft, Data curation, Investigation, Methodology. MD: Investigation, Supervision, Writing – review & editing, Conceptualization. EC: Conceptualization, Data curation, Funding acquisition, Investigation, Project administration, Supervision, Writing – review & editing. SC: Conceptualization, Data curation, Project administration, Supervision, Writing – review & editing.

## Supplementary material

Table S1 GAP index and disease staging for IPF patients.

Table S2 Clinical characteristics of IPF patients.

Table S3 2Multiple Categorical analysis with multiple hypothesis through Benjamini-Hochberg.

Supplementary File 1 Total proteins from Mass Spectrometry Analysis with LFQ ≠0 in at least one of the replicates.

Supplementary File 2 Total proteins from Mass Spectrometry Analysis with LFQ ≠0 in all the replicates after filtering for valid values.

Supplementary File 3 Total proteins from Mass Spectrometry after filtering for the protein distribution and PCA analysis.

Supplementary File 4 Total proteins from Mass Spectrometry grouping in CTRL, Stage I and Stage II.

Supplementary File 5 Total proteins from Mass Spectrometry after Z-score normalization and Volcano Plot building between CTRL and Stage I patient with T-Test, FDR: 0.05 and -0.5 <Fc< 0.5.

Supplementary File 6 Total proteins from Mass Spectrometry after Z-score normalization and Volcano Plot building between CTRL and Stage II patient with T-Test, FDR: 0.05 and -0.5 <Fc< 0.5.

Supplementary File 7 Total proteins from Mass Spectrometry after Z-score normalization and Volcano Plot building between CTRL and FVC<75 patient with T-Test, FDR: 0.05 and -0.5 <Fc< 0.5.

Supplementary File 8 Total proteins from Mass Spectrometry after Z-score normalization and Volcano Plot building between CTRL and DLco<55 patient with T-Test, FDR: 0.05 and -0.5 <Fc< 0.5.

Supplementary File 9 Multiple category Analysis with KRGG for the HeatMap showing differentially expressed proteins between CTRL, Stage I, Stage II.

Supplementary File 10 Network Analyst enriched Pathway on the biggest network through KEGG pathway.

## Glossary

**Table:**

LC-MS/MS	liquid chromatography-mass spectrometry
TGFβ	transforming growth factor β
FVC	forced vital capacity
DLco	Diffusion Lung carbon monoxide
PRDX2	peroxiredoxin2
LCP1	lymphocyte cytosolic protein 1
TAGLN2	transgelin 2
LUM	lumican
OGN	osteoglycin/mimecan
MUC5B	mucin 5B
STRING	search tool for retrieval interacting genes/protein
LFQ	label-free quantification
SLB	surgical lung biopsy
SH3BGLR3	SH3 Domain-Binding Glutamic Acid-Rich-Like Protein 1
PRDX3	peroxiredoxin 3
HBD	hemoglobin Subunit Delta
TUBB1	tubulin beta-1 chain
PTMS	parathymosin
STAT4	signal transducer and activator of transcription 4
GCA	grancalcin
TXN	thioredoxin
FLNA	filamin-A
COL1A2	collagen alpha-2(I) chain
COL3A1	collagen alpha-I(III) chain
ACAN	aggrecan core protein
SOD1	superoxide dismutase 1
KEGG	kyoto encyclopedia of genes and genomes
NSCLC	non-small cell lung cancer
CAF	cancer associated fibroblast
PI3K	phosphatidylinositol-3-kinase
MAPK	mitogen-activated protein kinase
