# Supplementary material for: Proteomic profiling of formalin-fixed paraffine-embedded tissue reveals key proteins related to lung dysfunction in idiopathic pulmonary fibrosis
Source: Front Oncol. 2024 Jan 23;13:1275346. doi: 10.3389/fonc.2023.1275346 (PMC10844556; doi:10.3389/fonc.2023.1275346)
Supplement: Table S1 — GAP index and disease staging for IPF patients. [file DataSheet_1.docx]

Supplementary Material

# Supplementary Data

# Supplementary Methods 1: Total proteins processing for mass spectrometry analysis and western blot

35 µg of total protein lysate for each sample was used for Western Blot. For mass spectrometry analysis, we added 0.4 ml methanol (cod.414816, CARLO ERBA, USA) to 0.1 ml of total protein extraction, previously obtained, 0.1 ml chloroform (cod.415154, CARLO ERBA, USA) and 0.3 ml RNA-ase, DNA-ase, protease free water and centrifuged at 9.000 rpm. After washing the pellet with 1 ml ethanol, the pellet after centrifugation was dissolved in 10 µl of 1% (w/v) Rapigest SF in 25mM NH_4_HCO_3_ (cod. 186001861, Waters, Milford, MA) and 10 µl 50 mM DTT in 25 mM NH_4_HCO_3_. After 1h incubation at 37°C with agitation at 950 rpm, samples were treated with 10 µl of 100 mM iodoacetamide (BioUltra. cod. 144-48-9, Sigma Aldrich, USA) in 25 mM NH_4_HCO_3_, incubated at 37°C for 1 h with agitation at 950 rpm and treated with 90 µl of 25 mM NH_4_HCO_3_. Then, 20 µl of Trypsin-grade (cod. V5111, Promega, WI, USA) was added (0.25 µg/µl) in 25 mM NH_4_HCO_3_ and incubated overnight at 37°C. After about 16 hours, the digestion was stopped by adding 20 µl of 5% (v/v) TFA (Trifluoroacetic Acid cod.102253211, Sigma Aldrich, USA) and incubated at 37°C for 1h with agitation at 950 rpm. The samples were centrifuged at 13.000 rpm for 30 minutes at room temperature and the supernatant containing the tryptic peptide were dried under vacuum using Speedvac evaporators.

# 2 Supplementary Methods 2: Experimental setting for Mass Spectrometry and data analysis

# An amount of peptide solution to have 50 µg on the column was injected into a Thermo Scientific Dionex Ultimate 3000 UHPLC coupled to a Thermo Ultrahigh-resolution Q Exactive mass spectrometer (Thermo Scientific, Bremen, Germany) HESI source was operated in positive mode with the following settings: spray voltage 4 kV, capillary temperature 320°C, sheat gas 55, auxiliary gas 30, spare gas 3, S-lens RF level 55, probe heater temp 300°C. The column, thermostatted at 30°C, was equilibrated with 0.5 mL/min of 0.1% formic acid in water (solvent A) with 2% solvent B (acetonitrile with 0.1% formic acid); after 0.5 minutes from the sample injection, solvent B was linearly increased from 2 to 28% in 150 minutes. From minutes 150.5 to 170.5, B% was raised to 98%, kept at 98% for 17.4 minutes and brought back to 2% B in 0.1 minutes for the reconditioning step. Each sample required a total run time of 200 minutes. Centroided MS and MS2 spectra were recorded from 200 to 2’000 in Full MS/dd-MS² (TOPN) mode, at a resolution of 35’000 and 17’500, respectively. The eight most intense ions were selected for MS^2^ nitrogen-promoted collision-induced dissociation (NCE = 28). Precursor dynamic exclusion (10 seconds) and apex triggering (0.2 to 15s) were set. The mass spectrometer was calibrated before the start of the analyses. A lock mass (419.3156, diisononyl phthalate [M+H]+) was monitored during the very first seconds of each analysis. Then, raw MS files were analyzed with the MaxQuant software version (v1.6.2.6) (Max Planck Institute, Martinsried, Germany)(Cox e Mann 2008) (Schaab et al. 2012) (Tyanova et al. 2015) against the Human Uniprot database (NCBI: txid9606, 2023_01, 245,871,724 sequence entries), including both the protein modifications such as carbamidomethylation (C) (fixed), oxidation (M) (variable) and N-terminal acetylation (variable) and the enzyme specificity set to trypsin; the maximum missed cleavages were set to 2 and the parent peptide masses searched with a maximal initial mass deviation of 10 p.p.m. The false discovery rate (FDR) filtration on the peptide spectrum was set to 0.01. Proteins were quantified using LFQ (Label-Free Quantification) intensity. Thus, proteins with LFQ ≠ 0 in all three replicates for each sample were subjected to bioinformatics analysis using the freely available software Perseus v1.6.15.0 (Max Planck Institute, Martinsried, Germany, www.perseus-framework.org)(Tyanova et al. 2016). Here, LFQ intensities were log_2_ transformed and there were retained only samples with normal data distribution. Results were then filtered to remove contaminants, reverse matches, proteins only identified by site and a MS/MS spectral count ≥2. Subsequently, data were row filtered according to valid values (minimum valid percentage, 75%) and then normalized by median subtraction. Then, a downward shift of 1.8, and a width of 0.3 standard deviations was set in the normal distribution for missing values. For the pairwise comparisons performed with the Volcano plot, the X-axis showed the fold change calculated by the difference between the average of log_2_ values (Δlog_2_ (LFQ intensity) of proteins detected in CTRL patients vs. proteins detected in stratified IPF patients. The related statistical analysis was performed with Student’s t-test for two-tailed unpaired data where the p-values were adjusted using the FDR-based permutation method (FDR: 0.01). Thus, the fold change FC ≥ 0.5 and ≤0.5 with a consistent p-value of 0.05 represented a cutoff for proteins differentially expressed.

# Supplementary Tables and Figures Legend

**Table S1**


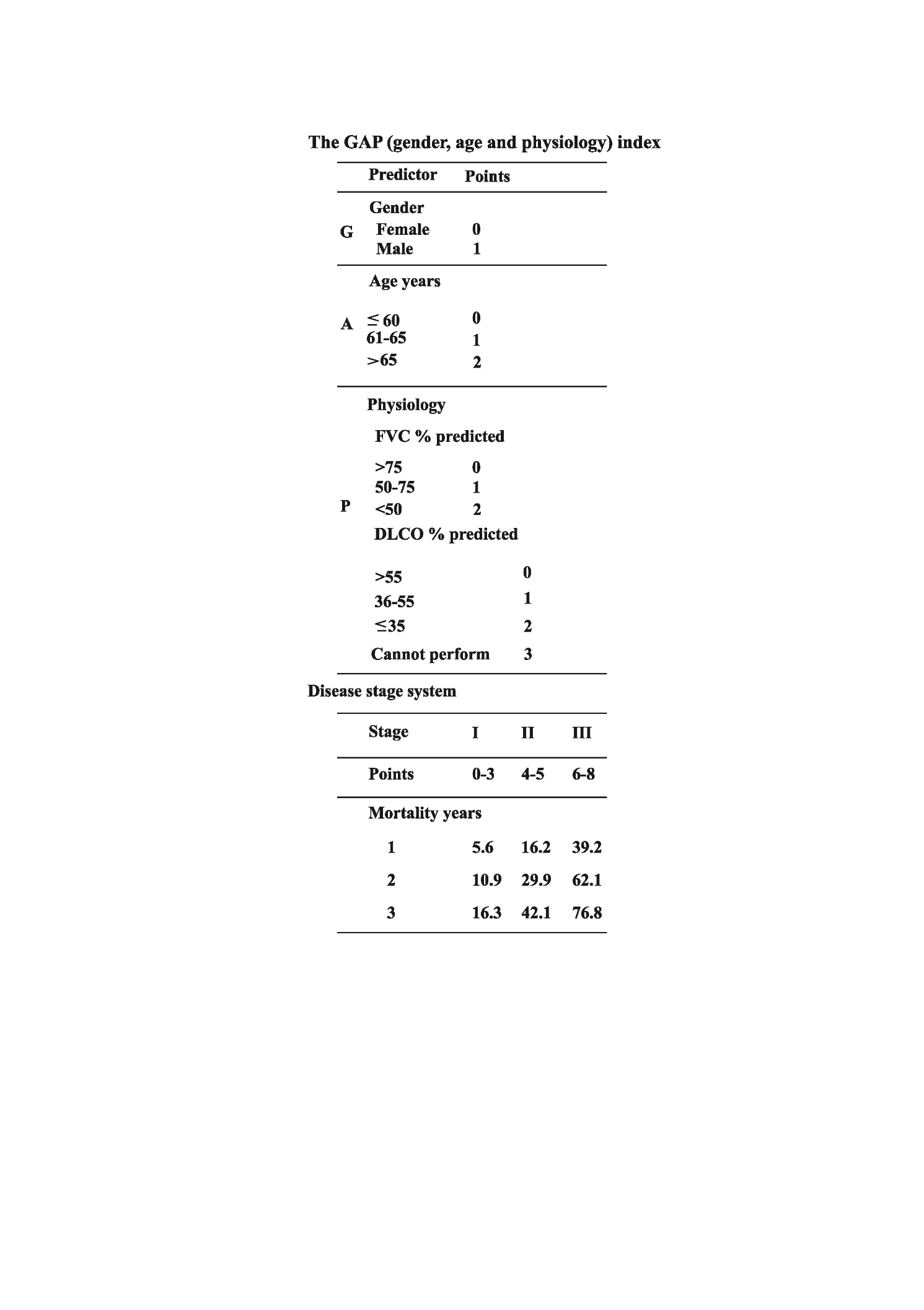


Table S1: GAP index and disease staging for IPF patients

**Table S2**

| **IPF Patient characteristics** | **Mean ± SD/n**  **n=11** | **Range/%** |
| --- | --- | --- |
| Age, years | 68.27±6.4 | 50–79 |
| Gender, male | 7 | 63.6 |
| Smoker, yes | 8 | 72.7 |
| GAP score 0-3  GAP score 4-6 | 7  4 | 63.6  36.3 |
| Stage I | 7 | 63.6 |
| Stage II | 4 | 36.3 |
| Stage III | 0 | 0 |
| Lung Physiology %  FVC pred at SLB | 90.6±17.22 | 61–123 |
| DLCO pred at SLB | 58.3±13.4 | 40–88 |

Table S2: Clinical characteristics of IPF patients selected for the study

| **Selection column** | **Selection value** | **Category column** | **Category value** | **Selection size** | **P value** | **Benj. Hoch. FDR** |
| --- | --- | --- | --- | --- | --- | --- |
| GOCC name | protein-DNA complex | KEGG name | Systemic lupus erythematosus | 3 | 0,094344 | 0.017904 |
| GOCC name | nucleosome | KEGG name | Systemic lupus erythematosus | 3 | 0,094344 | 0.017904 |
| GOCC name | hemoglobin complex | KEGG name | Malaria | 3 | 0,094344 | 0.017904 |
| GOCC name | collagen | KEGG name | Protein digestion and absorption | 7 | 0,0056834 | 0.0095757 |
| GOCC name | endoplasmic reticulum part | KEGG name | Protein digestion and absorption | 9 | 3.41E-06 | 0.0097072 |
| GOCC name | endoplasmic reticulum lumen | KEGG name | Protein digestion and absorption | 9 | 3.41E-06 | 0.0097072 |
| GOCC name | extracellular matrix part | KEGG name | Protein digestion and absorption | 9 | 3.41E-06 | 0.0097072 |
| GOCC name | collagen | KEGG name | ECM-receptor interaction | 7 | 0,11356 | 0.019397 |
| GOCC name | extracellular matrix part | KEGG name | ECM-receptor interaction | 9 | 0,011213 | 0.0095757 |
| GOCC name | extracellular matrix | KEGG name | ECM-receptor interaction | 16 | 0,019578 | 0.0097072 |

**Table S3**

Table S3: Multiple Categorical analysis with multiple hypothesis through Benjamini-Hochberg correction. The functional analysis together with the corresponding enrichment factor and p-value are shown: the ECM receptor interaction, protein-DNA complex, nucleosome, the hemoglobin complex, the focal adhesion and, protein digestion and absorption

## 3.1 Supplementary Figures Legend

**Supplementary Figure 1:** Flow chart summarizing the mass spectrometry analysis that shows the inclusion and exclusion criteria of samples deriving from patients with IPF and control lung tissues.

**Supplementary Figure 2:** Multi-scatter plot of total triplicate for each sample analyzed and the corresponding Pearson’s correlation

**Supplementary Figure 3**: Volcano plot showed both up-regulated (left) and down-regulated (right) proteins in the pairwise comparison between StageI and StageII patients. Volcano plots show log 2 FC (x-axis) and −log10 value of p-value (y-axis). The thresholds are set for a base log 2 > 0.5 and FDR p value < 0.05.

**Supplementary Figure 4:** Immunohistochemical analysis performed on FFPE slides of Patient 1 (Stage I, FVC>75, DLCO>55) and Patient 11 (Stage II, FVC>75, DLCO<55) by using the specific antibodies for TAGLN2 (A), PRDX2 (B), LCP-1 (C), LUM (D), OGN (E), 20X objective, scale bar, 20 µm.

**Supplementary Figure 5:** Protein-protein interaction analysis though GeneNetwork Analyst. Protein-protein interaction (PPI) networks was built with GeneNetwork Analyst software. By adding in the analysis 20 other proteins that were not statistically significant but differentially expressed between CTRL and Stage II patients a second order network was performed based on the literature curated comprehensive data that showed one complex sub-network of about 643 nodes and 870 edges were LCP1, PRDX2, OGN and TAGLN2 linked together with other molecular interactors. The blue nodes highlight the molecular interactor involved in pathways in cancer (KEGG) which is the most enriched pathway in this sub-network.

**Supplementary Figure 6:** Second complex sub-network of a second order network based on the literature curated comprehensive of about 4 nodes and 3 edges were LUM linked together with other molecular interactors.

**Supplementary Figure 7:** Original membranes of the Western Blot performed to validate the differentially expressed proteins referred to the main Figure 4A (A) Western blot representing from top to bottom the COL1A1, β-Actin, TAGLN2 antibodies and SH3BGLR3 (not shown in the main results) in Wcl_293T cells as control of western blot, CTRL2, CTRL3 and CTRL1 patient, patients 1 and 4 (p1 and p4_Stage I), p9 patient (p9_Stage II) and p8 and p11 IPF patients (Stage II) in the second original membrane on the right. (B) pSMAD (C) LCP1 and PRDX2 from top to bottom (D) OGN (arrow) and (E) lumican (arrow) after membrane stripping are shown for the same original membranes. (D) and (E) present a single band at 70 KDa which represents the previous signal of LCP-1 not completely stripped.

**Supplementary Figure 8:** Original membranes of the Western blot of the main Figure 4B. (A) shows from the top to the bottom the COL1A1, the β-Actin and TAGLN2 signals for CTRL 4, 5,6 and CTRL7 patients and patients 12,13,14 and 15 with IPF (p12, p13, p14 and p15). (B) from the top to the bottom are shown the signals of the LCP-1 and the PDX2 antibodies. (C) LUM and the (D) OGN signals.
