# Supplementary figures and images for: Proteomic profiling of formalin-fixed paraffine-embedded tissue reveals key proteins related to lung dysfunction in idiopathic pulmonary fibrosis
Source: Front Oncol. 2024 Jan 23;13:1275346. doi: 10.3389/fonc.2023.1275346 (PMC10844556; doi:10.3389/fonc.2023.1275346)

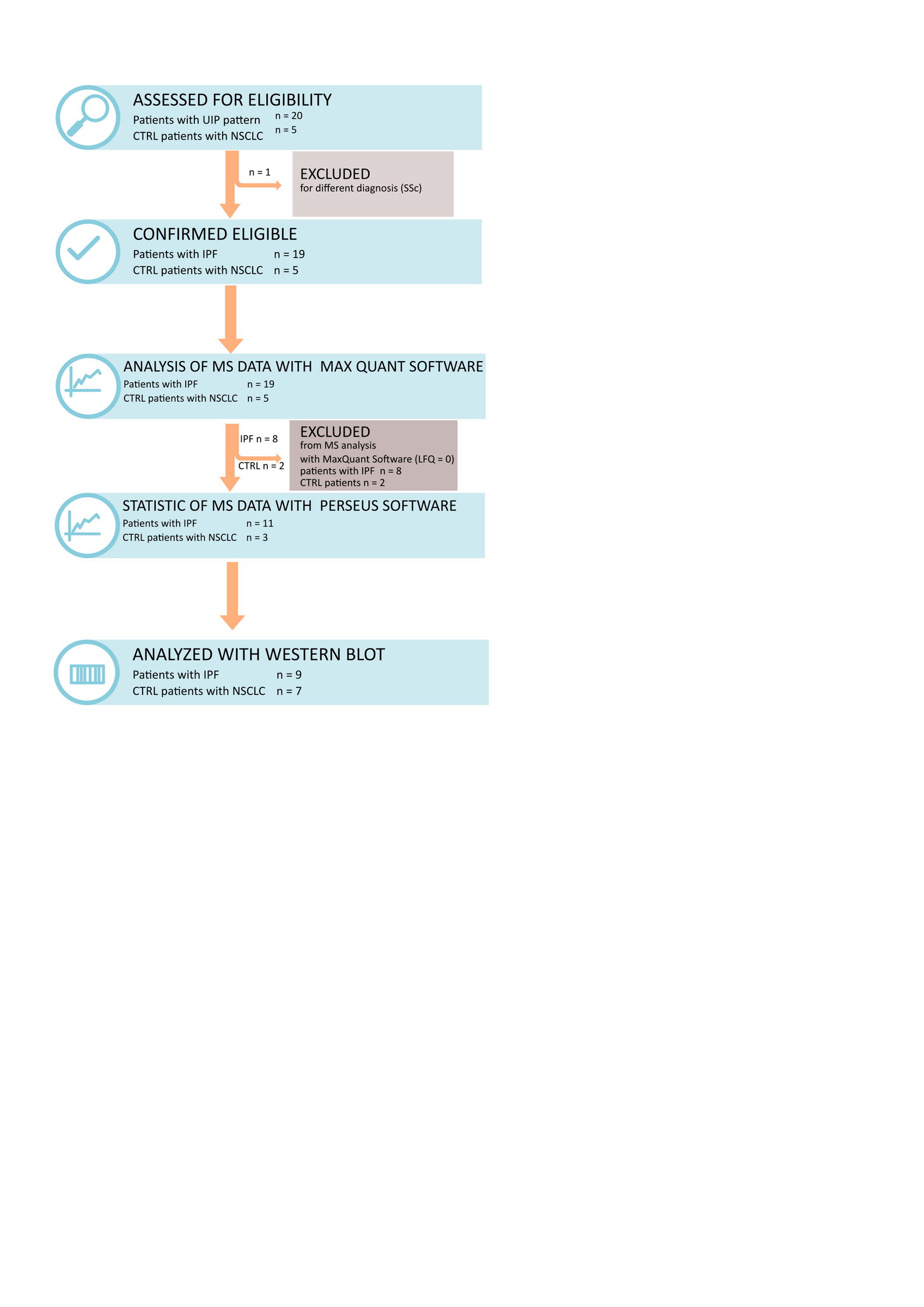

Supplement: Supplementary file 2 [file Image_1.tif]

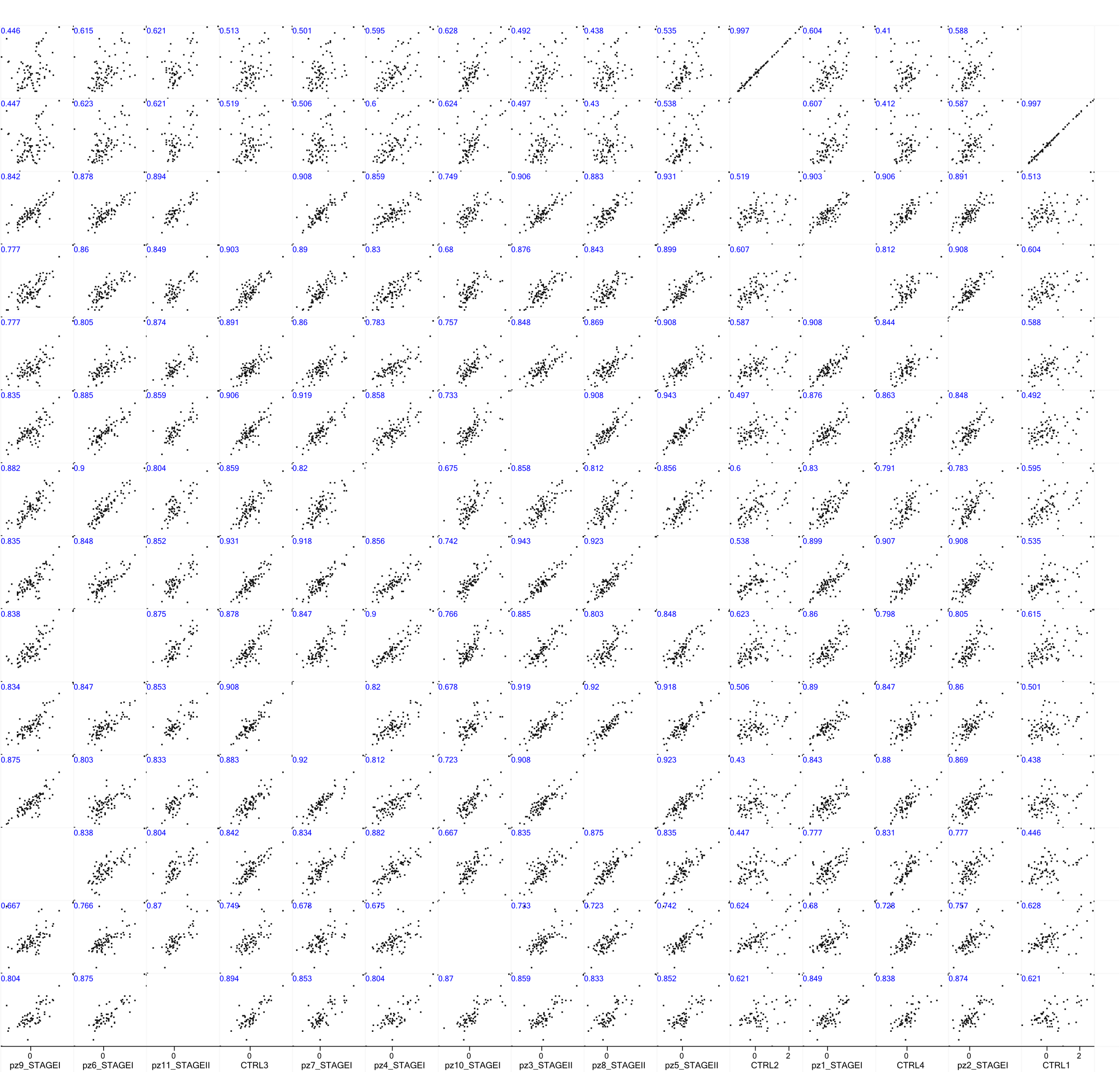

Supplement: Supplementary file 3 [file Image_2.tif]

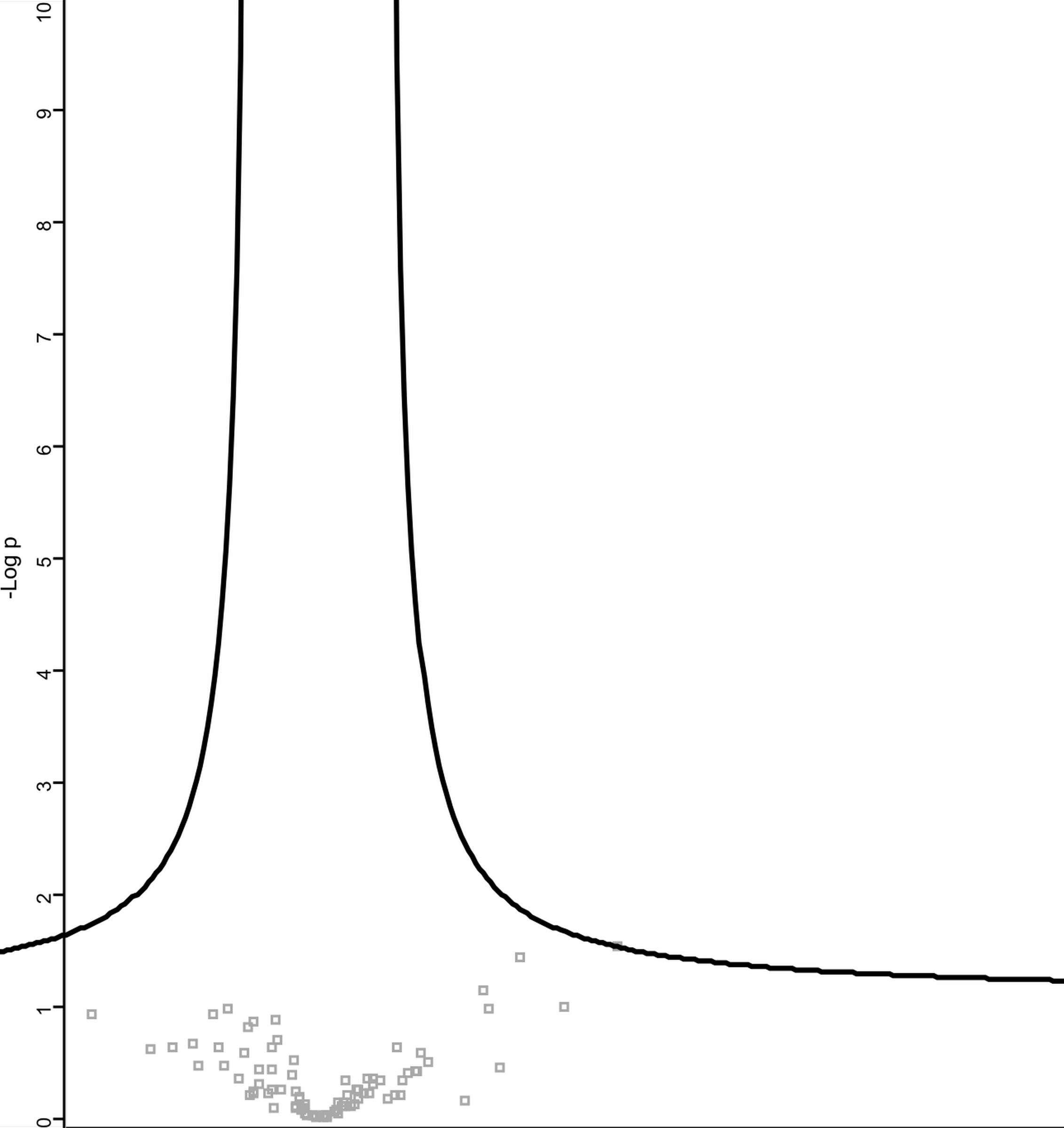

Supplement: Supplementary file 4 [file Image_3.tif]

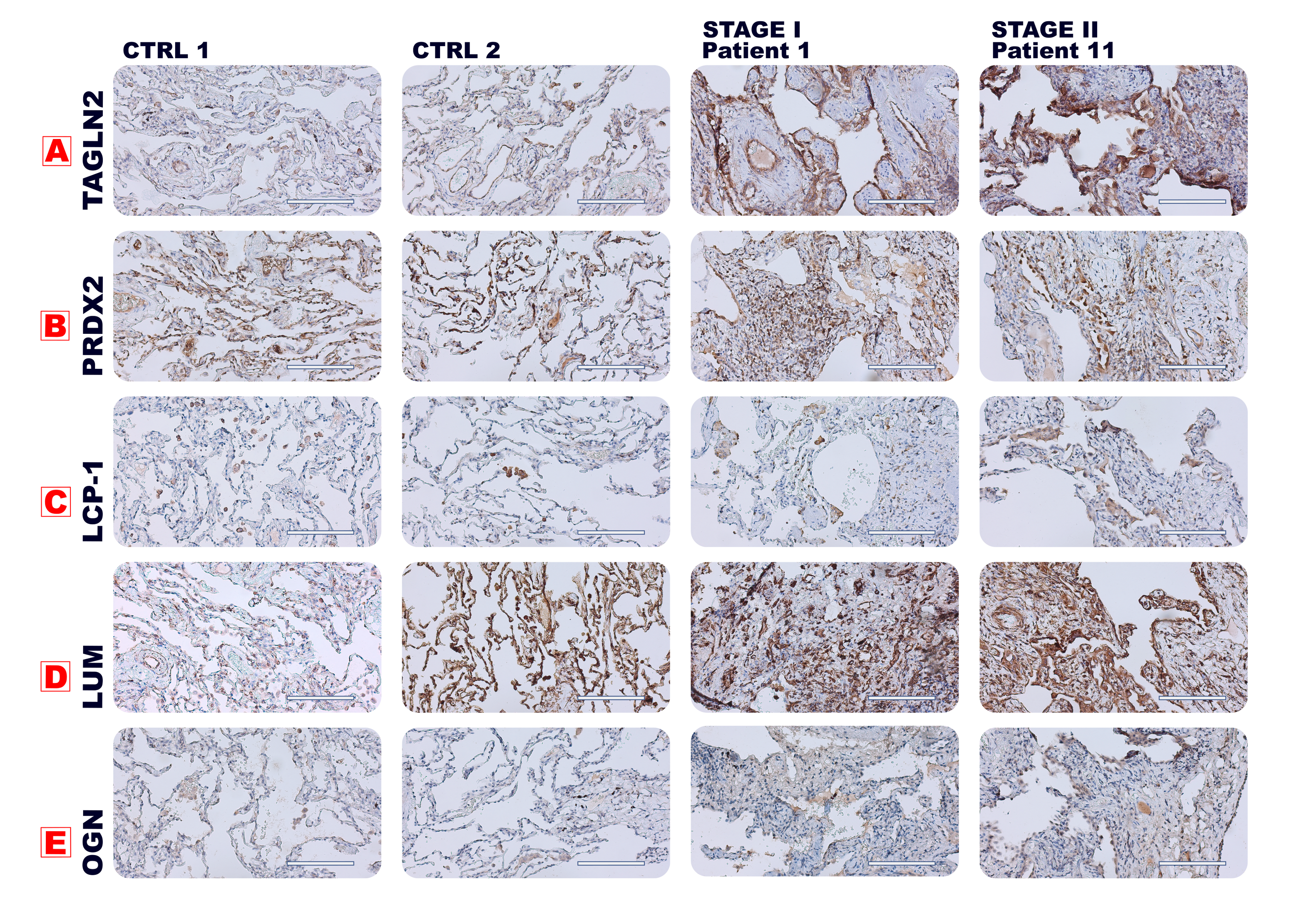

Supplement: Supplementary file 5 [file Image_4.tif]

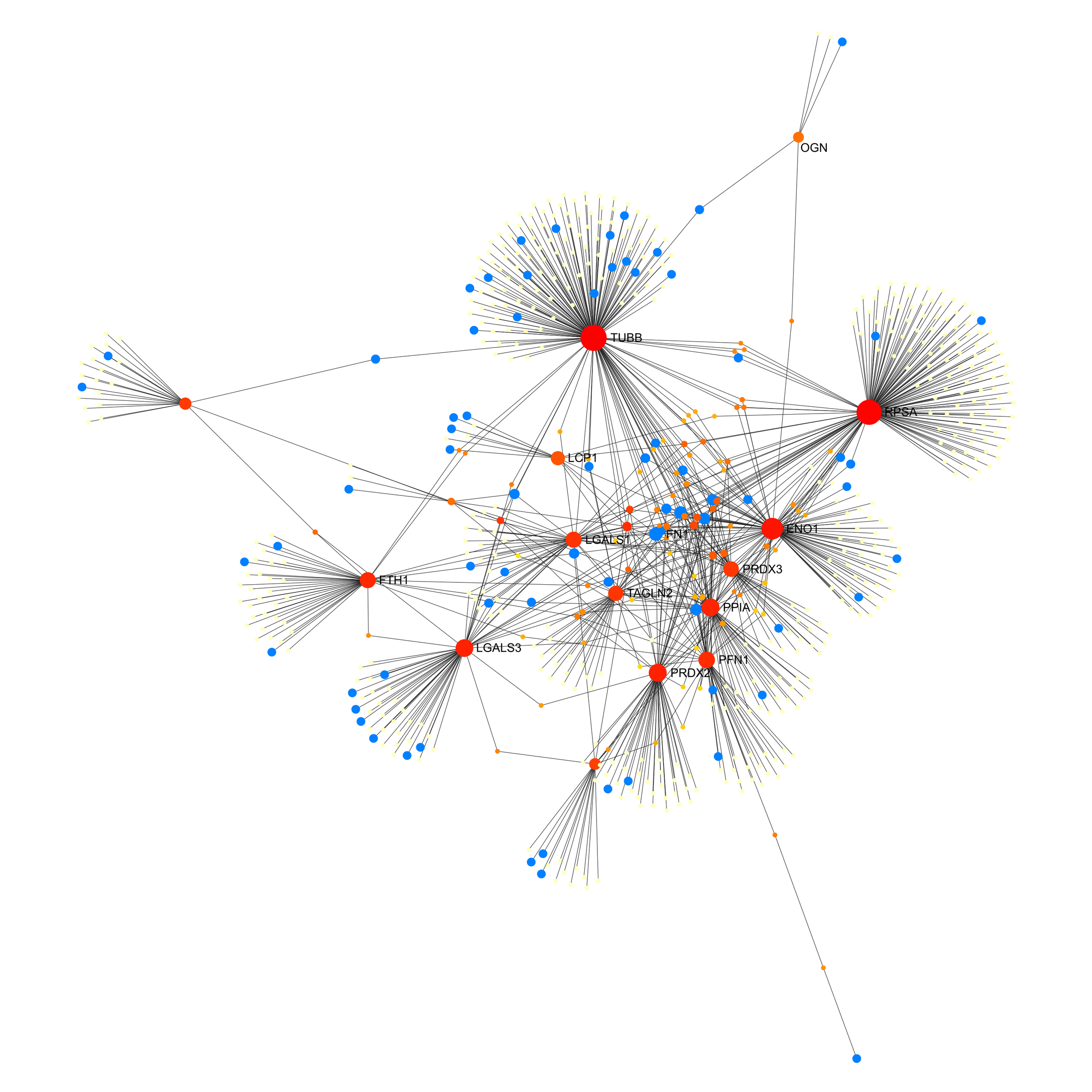

Supplement: Supplementary file 6 [file Image_5.tif]

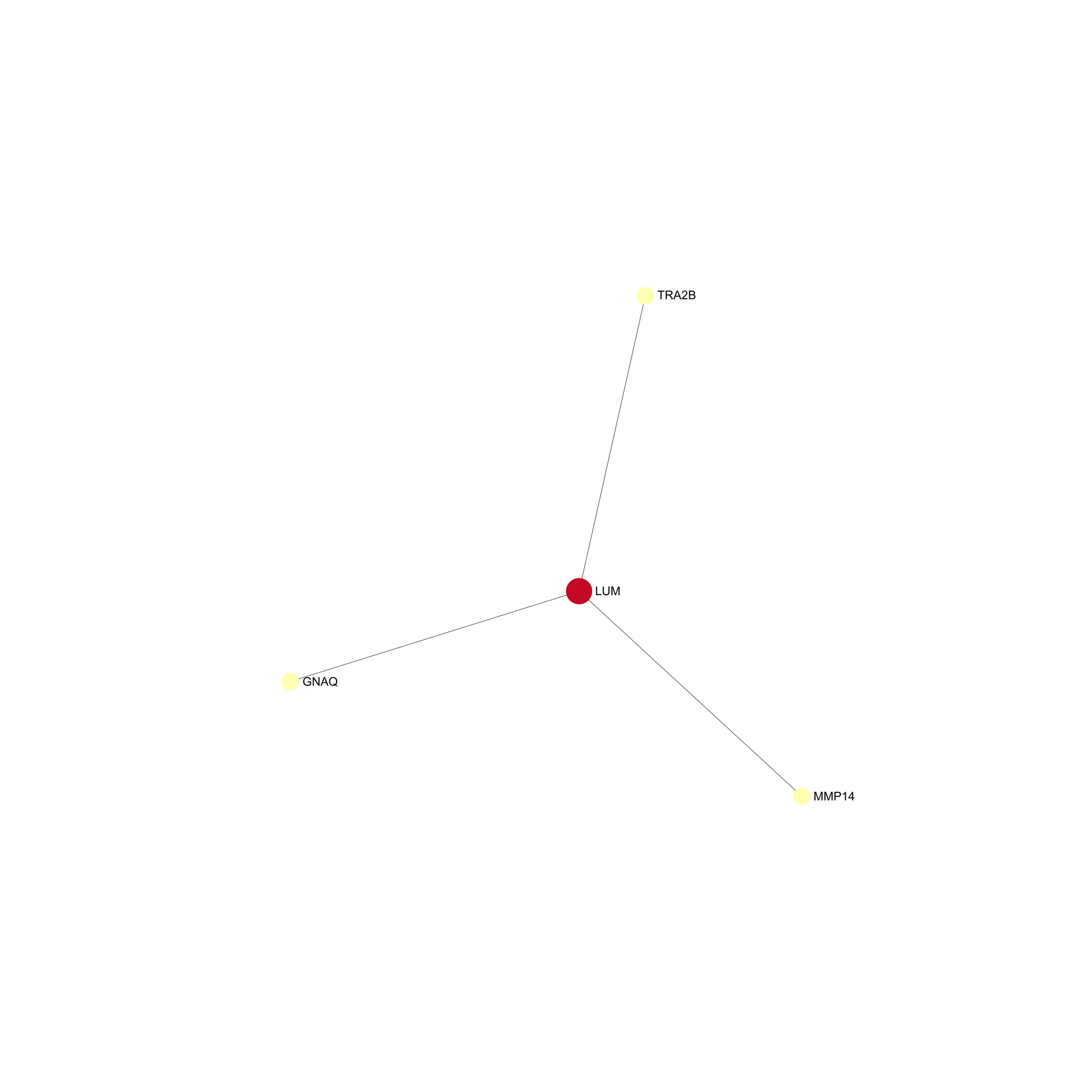

Supplement: Supplementary file 7 [file Image_6.tif]

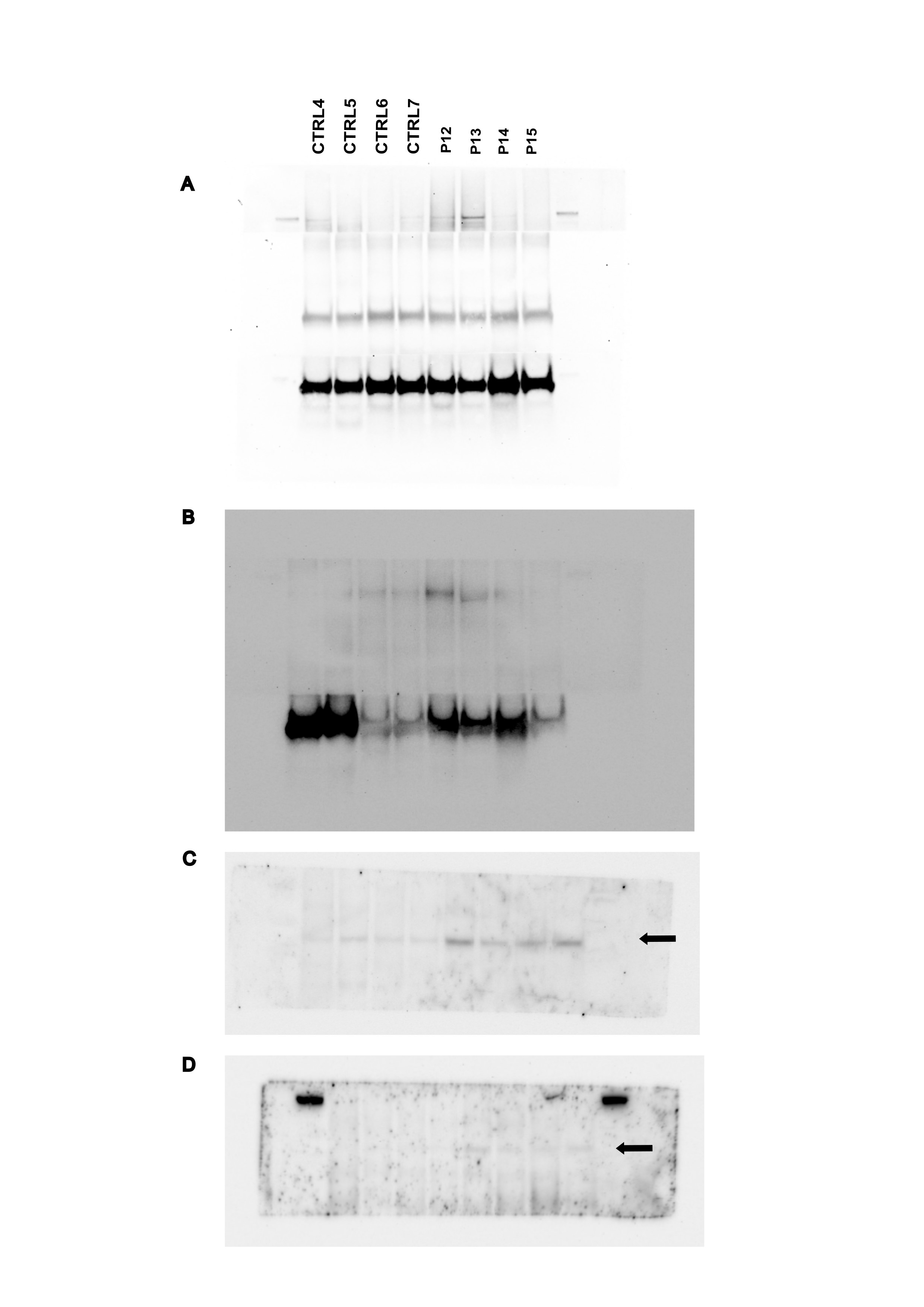

Supplement: Supplementary file 8 [file Image_7.jpeg]

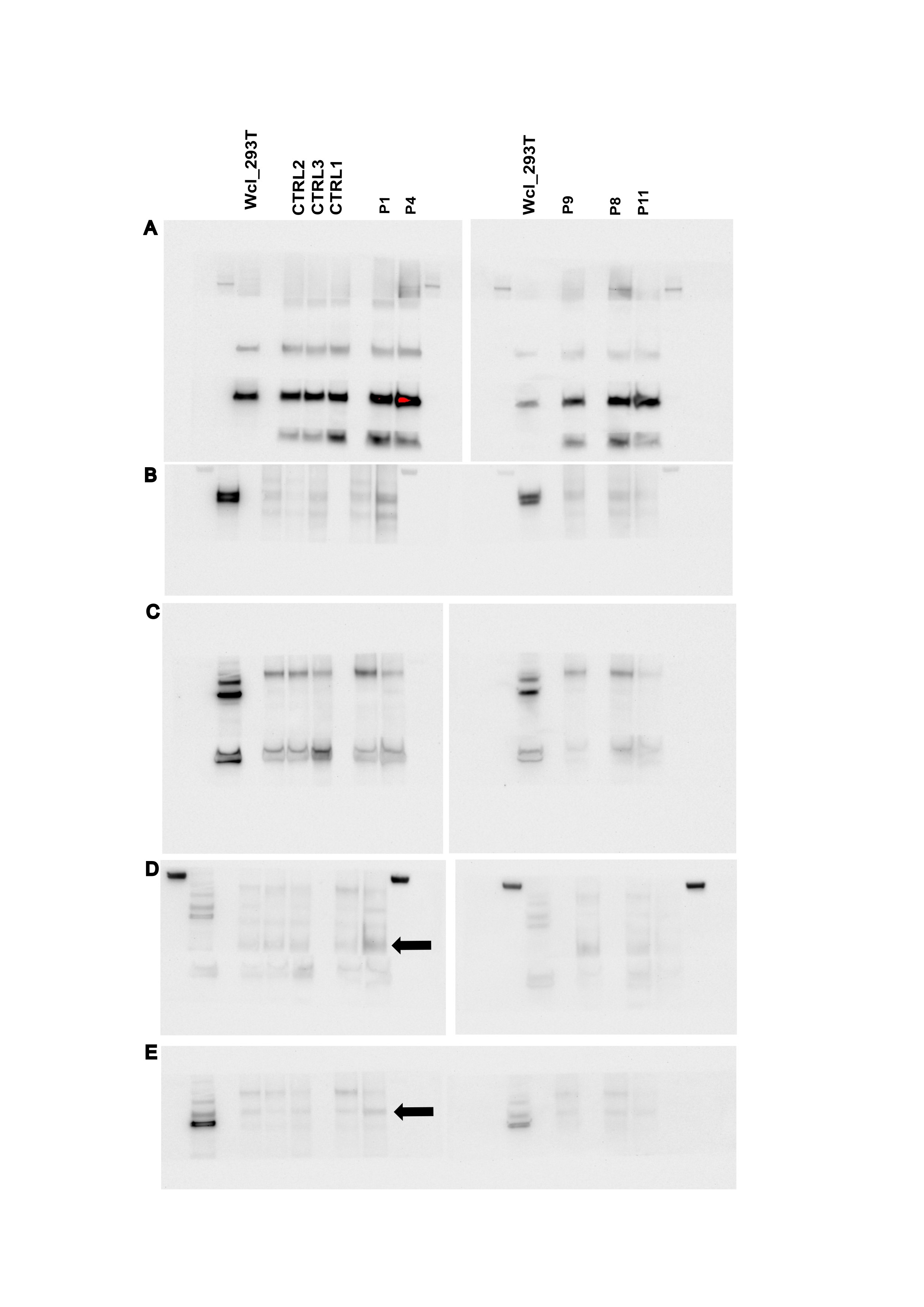

Supplement: Supplementary file 9 [file Image_8.jpeg]
